# Brief Temporal Perturbations in Somatosensory Reafference Disrupt Perceptual and Neural Attenuation and Increase Supplementary Motor Area–Cerebellar Connectivity

**DOI:** 10.1523/JNEUROSCI.1743-22.2023

**Published:** 2023-07-12

**Authors:** Konstantina Kilteni, Christian Houborg, H. Henrik Ehrsson

**Affiliations:** ^1^Department of Neuroscience, Karolinska Institutet, SE-171 77 Stockholm, Sweden; ^2^Donders Institute for Brain, Cognition and Behaviour, Radboud University, 6525 GD Nijmegen, The Netherlands

**Keywords:** cerebellum, motor prediction, somatosensory attenuation, somatosensory reafference, supplementary motor area, temporal perturbation

## Abstract

Intrinsic delays in sensory feedback can be detrimental for motor control. As a compensation strategy, the brain predicts the sensory consequences of movement via a forward model on the basis of a copy of the motor command. Using these predictions, the brain attenuates somatosensory reafference to facilitate the processing of exafferent information. Theoretically, this predictive attenuation is disrupted by (even minimal) temporal errors between the predicted and actual reafference; however, direct evidence of such disruption is lacking as previous neuroimaging studies contrasted nondelayed reafferent input with exafferent input. Here, we combined psychophysics with functional magnetic resonance imaging to test whether subtle perturbations in the timing of somatosensory reafference disrupt its predictive processing. Twenty-eight participants (14 women) generated touches on their left index finger by tapping a sensor with their right index finger. The touches on the left index finger were delivered close to the time of contact of the two fingers or with a temporal perturbation (i.e., 153 ms delay). We found that such a brief temporal perturbation disrupted the attenuation of the somatosensory reafference at both the perceptual and neural levels, leading to greater somatosensory and cerebellar responses and weaker somatosensory connectivity with the cerebellum, proportional to the perceptual changes. We interpret these effects as the failure of the forward model to predictively attenuate the perturbed somatosensory reafference. Moreover, we observed increased connectivity of the supplementary motor area with the cerebellum during the perturbations, which could indicate the communication of the temporal prediction error back to the motor centers.

**SIGNIFICANCE STATEMENT** Our brain receives somatosensory feedback from our movements with a delay. To counteract these delays, motor control theories postulate that the brain predicts the timing of somatosensory consequences of our movements and attenuates sensations received at that time. Thus, a self-generated touch feels weaker than an identical external touch. However, how subtle temporal errors between the predicted and actual somatosensory feedback perturb this predictive attenuation remains unknown. We show that such errors make the otherwise attenuated touch feel stronger, elicit stronger somatosensory responses, weaken cerebellar connectivity with somatosensory areas, and increase this connectivity with motor areas. These findings show that motor and cerebellar areas are fundamental in forming temporal predictions about the sensory consequences of our movements.

## Introduction

During voluntary movement, our sensorimotor loop suffers from ubiquitous delays because of sensory transduction, neural conduction, and brain processing of the sensory feedback (Wolpert and Flanagan, 2001; Franklin and Wolpert, 2011). These delays have a nonnegligible magnitude, even exceeding ∼100 ms (Scott, 2016), and their impact can be detrimental, destabilizing our motor output and leading to oscillatory movements when rapidly correcting motor errors (Miall and Wolpert, 1996; Kawato, 1999). To compensate for the delayed feedback, the brain uses a forward model in combination with a copy of the motor command (efference copy) to predict sensory consequences of the movement and thus relies less on the delayed input (Shadmehr et al., 2010; McNamee and Wolpert, 2019). These predictions allow to prospectively correct the motor command in case of errors (Shadmehr et al., 2010) and improve the estimation of the current state of our body (Todorov and Jordan, 2002; Scott, 2004; Shadmehr et al., 2008).

The forward-model-based predictions further serve to differentiate sensory reafference from exafference. Both animal and human studies have repeatedly shown that signals received at the predicted time, and thus corresponding to sensory consequences of the movement, are suppressed to facilitate the processing of external signals (Blakemore et al., 2000a; Brooks and Cullen, 2019; McNamee and Wolpert, 2019; Audette et al., 2022; Kilteni, 2023). For example, when the right hand is used to touch the left hand, reafferent touches of the left hand feel systematically weaker (Blakemore et al., 1999; Shergill et al., 2003; Kilteni and Ehrsson, 2017a,b, 2022; Kilteni et al., 2018, 2020; Asimakidou et al., 2022; Job and Kilteni, 2023; Timar et al., 2023) and elicit weaker somatosensory responses than exafferent touches of identical intensity (Blakemore et al., 1998; Hesse et al., 2010; Kilteni and Ehrsson, 2020). Critically, this attenuation of sensory reafference is time locked to the expected timing of feedback, and it is reduced, or even absent, when identical somatosensory input is presented earlier (Bays et al., 2005) or later (Blakemore et al., 1999; Bays et al., 2005; Kilteni et al., 2019, 2021).

From a theoretical perspective, the cerebellum (CB) implements the forward model and predicts the sensory consequences of movements (Shadmehr et al., 2008; McNamee and Wolpert, 2019; Popa and Ebner, 2019). It uses the efference copy, which is possibly generated in premotor areas (Voss et al., 2006; Christensen et al., 2007; Pynn and DeSouza, 2013), including the supplementary motor (SMA) area (Haggard and Whitford, 2004), to attenuate the reafferent somatosensory input. These computational processes are very sensitive to errors between the predicted and actual sensory feedback (Wolpert and Flanagan, 2001; Shadmehr et al., 2010); under certain conditions, errors can force the sensorimotor system to either refine its motor plan (Johnson et al., 2019), reoptimize the predictions of the forward model after systematic exposure to the errors (Izawa et al., 2008), or disregard them and attribute them to external causes if these errors are large (Wei and Körding, 2009; Wilke et al., 2013). Previous human neuroimaging studies have manipulated the timing of somatosensory feedback by imposing large temporal delays (reaching 400–500 ms; Blakemore et al., 2001; Shergill et al., 2013). However, the brain might have treated these long temporal errors as exafferent somatosensory input (i.e., externally generated) rather than delayed reafference input, and these large delays might not have interfered with the theorized predictive processes at the behavioral or neural levels (i.e., the long delays might not have been treated as temporal errors in the prediction of the somatosensory feedback). Therefore, how subtle temporal perturbations in somatosensory reafference disrupt its predictive processing remains unknown. Moreover, none of these earlier neuroimaging experiments assessed the effect of delays on brain activity and perception within the same study. Consequently, whether and how perceptual and neural effects elicited by temporal perturbations are related is not well understood.

By combining psychophysics with functional magnetic resonance imaging (fMRI), we investigated perceptual and neural responses to the presence (50% trials) or absence (50% trials) of brief temporal perturbations between right-hand movements and somatosensory feedback of the left hand. Specifically, trials with a 153 ms temporal perturbation were compared with nonperturbed trials with a minimal delay of 53 ms, which was the smallest delay our system could achieve. Brief temporal perturbations on the order of 100–200 ms are not typically detectable (Blakemore et al., 1999) and do not lead to sensorimotor adaptation unless they are persistently presented (Kilteni et al., 2019). However, these brief temporal perturbations of ∼150 ms should theoretically disrupt the sensorimotor loop in two ways. First, these delays should interrupt the attenuation of the somatosensory reafference by the forward model, leading to greater somatosensory and cerebellar responses and weaker somatosensory connectivity with the cerebellum. Second, they should increase the connectivity of the supplementary motor area with the cerebellum to convey the error to the motor centers in line with computational models of sensorimotor control (Miall and Wolpert, 1996; Johnson et al., 2019). In contrast, trials where the touch is delivered close to its predicted timing, that is, with a 53 ms delay, are expected to have no impact on somatosensory attenuation (Bays et al., 2005) or the predictive mechanisms and simulate natural self-touch.

## Materials and Methods

### Participants

After providing written informed consent, 29 volunteers (15 women, 14 men; 27 right-handed, 2 ambidextrous) age 19–38 years participated in the study. Handedness was assessed using the Edinburgh Handedness Inventory (Oldfield, 1971). The sample size was set to 30 based on our previous study (Kilteni and Ehrsson, 2020), but because of scanner technical issues, fMRI data were collected from only 29 individuals. After data collection, one participant was further excluded for giving the same response to almost all trials (49 of 50 trials) in one of the two conditions of the psychophysical task, making the psychophysical modeling unreliable. For consistency, this participant was also excluded from the fMRI analysis. Therefore, both behavioral and fMRI analyses included data from a total of 28 participants (14 women, 14 men; 26 right-handed, 2 ambidextrous; 19–38 years old).

### Psychophysics and fMRI

The fMRI scan was conducted before the psychophysics session. We chose not to perform the psychophysics task during the fMRI scan to reduce participant movement during the scan (as necessary for task responses) and to ensure that the BOLD signal reflected activation related to the sensorimotor task and not to decision-making, working memory, or other cognitive processes involved in the psychophysics task (see below). The psychophysics experiment was conducted in the MR scanner environment using the same equipment (same motor setup and force sensors) as used in the fMRI session (see below). After the fMRI experiment and the psychophysics session, additional fMRI runs and psychophysical tasks were conducted as part of a different study addressing a separate question (data not shown). The Swedish Ethical Review Authority approved the study (project #2016/445-31/2, amendment #2018:1397-32).

### Procedures and experimental design for the psychophysical task

**Figure 1.**
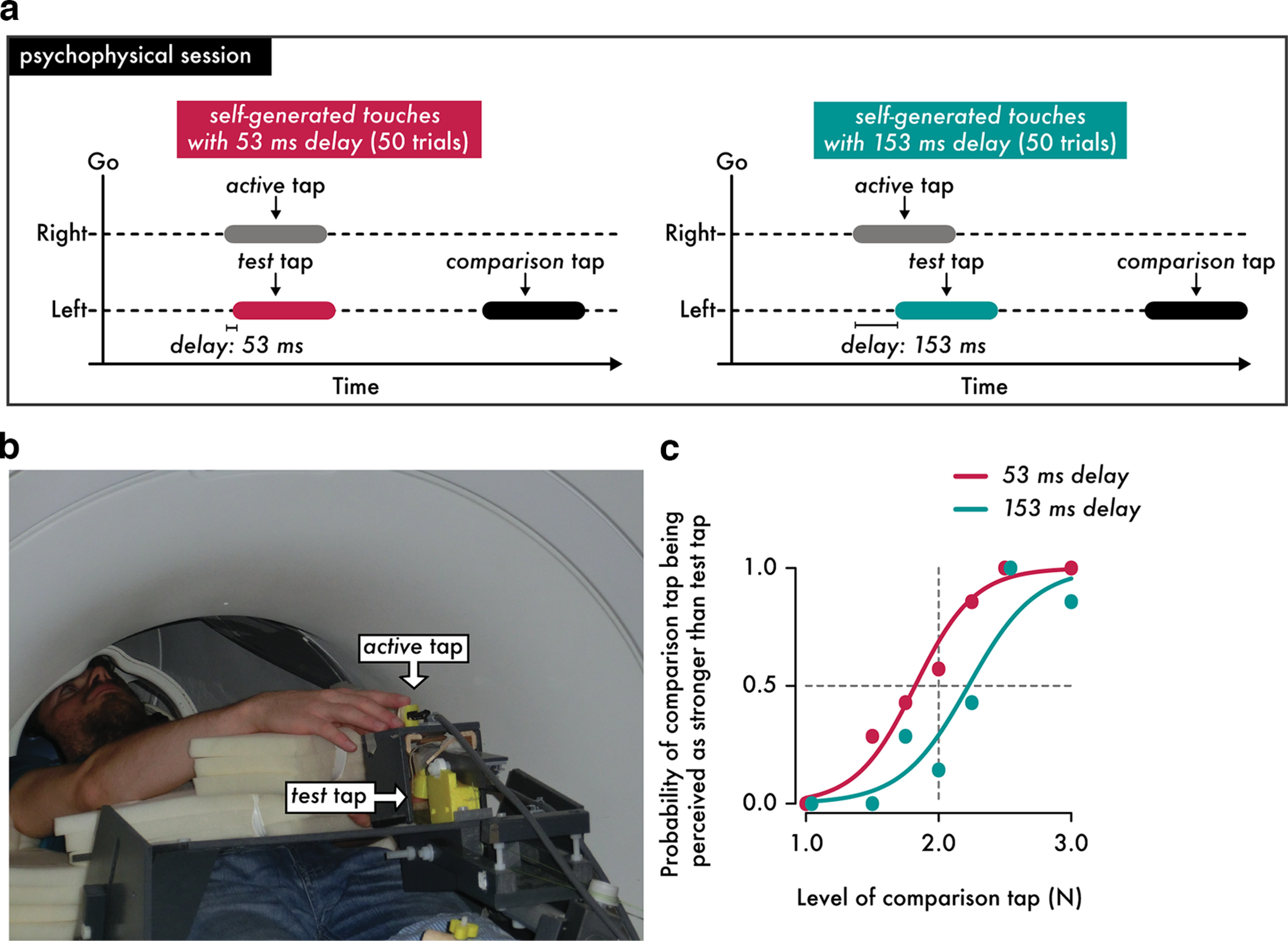
The psychophysical session conducted inside the MR scanner. ***a***, Participants performed a force discrimination task to assess their perceived magnitude of self-generated touches of 250 ms duration with a 53 ms or 153 ms delay. In this task they received one of two taps (the test or the comparison tap) on the pulp of their left index finger from an electric motor. The participants administered the test tap on their left index finger by actively tapping a sensor with their right index finger (active tap, gray rectangle); this test tap was received with a 53 ms delay (left, intrinsic delay of the fMRI setup, magenta rectangle) or with a 153 ms delay (right, cyan rectangle). Next, a second tap (comparison tap) of variable magnitude (black rectangle) was applied to their left index finger, and participants verbally reported which of the two taps (i.e., the test or the comparison tap) felt stronger. ***b***, Overview of the fMRI-compatible setup used in the psychophysics experiments and in the fMRI experiment. The psychophysics task was performed while the subjects were lying on the scanner bed without being scanned. ***c***, Responses and fitted logistic models of the responses of one representative participant in the two experimental conditions. The two partially overlapped data points are horizontally jittered to avoid complete overlap.

The psychophysical task was a two-alternative forced-choice force-discrimination task (Fig. 1*a*) that has been extensively used to assess somatosensory attenuation in previous studies (Bays et al., 2005, 2006; Kilteni et al., 2019, 2020, 2021; Asimakidou et al., 2022; Kilteni and Ehrsson, 2022); this task was used to quantify the perceived intensity of self-generated touches with the 53 ms delay and self-generated touches with the 153 ms delay. The duration of the temporal perturbation, that is, 153 ms, was chosen based on previous studies showing that it was long enough to perturb somatosensory attenuation (Blakemore et al., 1999; Bays et al., 2005; Kilteni et al., 2019). The 53 ms delay was the minimal intrinsic delay that would be produced by the current setup. Participants lay comfortably in a supine position on the MRI scanner bed. Their left hands were placed palms up on an MR-compatible plastic table with their left index finger in contact with a 3D-printed probe that contained a force sensor and was controlled by a motor through string-based transmission. Their right index finger was placed next to a second force sensor that was also placed on the table on top of (but not in contact with) the probe on the left index finger (Fig. 1*b*). Both arms were supported by sponges to maximize the comfort of the participants.

During the task, the participants were asked to tap the force sensor (active tap) with their right index finger after an auditory Go cue. Before the task, we instructed the participants to tap the sensor with their right index finger at an intensity comfortable for them and to use the same style of taps throughout the session. The active tap of the right index finger (force exceeding >0.4 N) was used to trigger the test tap on their left index finger after 53 ms or 153 ms. The test tap had a fixed intensity of 2 N. After a random delay between 800 and 1500 ms, participants received a subsequent externally generated tap (comparison tap) of variable intensity (1, 1.5, 1.75, 2, 2.25, 2.5, or 3 N). The taps were applied for ∼250 ms (mean ± SEM, 249.209 ± 5.146 ms). Participants were asked to verbally indicate which tap (the test or the comparison tap) felt stronger on their left index finger. Each condition consisted of 50 trials, where each level of the comparison tap was repeated seven times, except for the level of 2 N, which was repeated eight times. We opted to have a round number of trials (50) per condition; therefore, the extra repetition was allocated to the intermediate level (2 N) that corresponded to the intensity of the test tap. Consequently, there were 100 trials per participant. The order of conditions was randomized across participants. On average, participants administered an active tap of (mean ± SEM) 2.328 ± 0.203 N with their right index finger and received a test tap of 1.997 ± 0.004 N on their left index finger. The mean duration of the active tap produced by the participants was ∼180 ms (mean ± SEM, 176.432 ± 10.888 ms), whereas the duration of the test tap produced by the setup was 250 ms, as mentioned earlier.

As mentioned, the intrinsic delay of the system (i.e., the time difference between the active tap exceeding 0.4 N until the test tap reached 80% of its maximum magnitude) was ∼53 ms. Delays of the size of our intrinsic delay (∼50 ms) have been previously shown to not have an impact on somatosensory attenuation compared with smaller delays (e.g., 11 ms; Bays et al., 2005) suggesting that our self-generated touch with 53 ms delay simulates natural self-touch well. Moreover, our study was designed to compare self-generated touch conditions with the 53 ms or 153 ms delay, and thus the key comparison concerns the relative differences in perceptual and neural attenuation effects between these two otherwise equivalent active conditions.

### Processing, hypotheses, and statistical analysis of psychophysical data

No participants had missing trials in either of the two conditions, resulting in a total of 2800 trials (28 × 50 × 2 = 2800 trials). After data collection, we excluded any psychophysical trials in which the participants did not tap the sensor with their right index finger after the Go cue, tapped too lightly to trigger the touch on the left index finger (active tap <0.4. N), tapped more than once, or tapped before the Go cue as well as any trials in which the test tap was not applied correctly (test tap <1.85 N or test tap >2.15 N). This resulted in the exclusion of 117 trials of 2800 psychophysical trials (4.18%).

We fitted the participants' responses with a generalized linear model (Fig. 1*c*) using a logit link function as follows:

$$p=\frac{e^{\beta 0+\beta 1x}}{1+e^{\beta 0+\beta 1x}}.$$

We extracted two parameters of interest, the point of subjective equality (PSE), $PSE=-\frac{\beta 0}{\beta 1}$, which represents the intensity at which the test tap felt as strong as the comparison tap (p=0.5) and quantifies the perceived intensity of the test tap, and the just noticeable difference (JND), $JND=\frac{\text{log}(3)}{\beta 1}$, which reflects the participants' discrimination capacity. The PSE and JND are independent sensory judgments; higher PSE values indicate a stronger perceived magnitude, whereas higher JND values indicate a lower force discrimination capacity (i.e., lower somatosensory precision).

Based on previous studies (Blakemore et al., 1999; Bays et al., 2005; Kilteni et al., 2019, 2021), we expected to find a significant difference between the PSE values of the two conditions, with the self-generated touch with the 153 ms delay condition yielding a greater magnitude of the perceived touch than the self-generated touch with the 53 ms delay condition because of the temporal perturbation. We expected not to find any differences in the discrimination capacity (JND) between the two conditions, given our previous results involving the same right index finger movement and touch on the left index finger (Asimakidou et al., 2022; Kilteni and Ehrsson, 2022). Psychophysical data were analyzed using R (https://www.r-project.org/) and JASP (https://jasp-stats.org/) software. Data normality was assessed using the Shapiro–Wilk test, and planned comparisons were made using parametric analyses (paired *t* tests) given that the data were normally distributed. For each test, 95% confidence intervals (CI^95^) are reported. Effect sizes are given by Cohen's *d*. A Bayesian factor analysis was conducted for nonsignificant statistical comparisons of interest (default Cauchy priors with a scale of 0.707) to provide information about the level of support for the null hypothesis compared with the alternative hypothesis (*BF*_01_). Correlations between perceptual and neural responses (see below) were assessed with Kendall (*tau-b*) or Pearson (ρ) correlation coefficients depending on the normality of data distributions. All statistical tests were two tailed.

### Complementary post hoc psychophysical analysis

We performed a control analysis to test for the absence of any significant learning effects because of repeated exposure to the 153 ms delay in the self-generated touch with the 153 ms delay condition. According to one of our previous studies (Kilteni et al., 2019), the earliest significant learning of a 100 ms delay requires >400 exposure trials (50 initial exposure trials and 350 re-exposure trials (Kilteni et al., 2019, their experiment 2). Here, participants were exposed to only 50 trials in total during the psychophysical assessment; thus, no learning should be observed. However, if adaptation occurred, it could reduce the effect of brief temporal perturbations on psychophysical responses, especially by the end of the psychophysical task. To confirm the absence of such adaptation to delays, we fitted the participants' responses in the self-generated touch with the 53 ms delay and self-generated touch with the 153 ms delay conditions separately for the first and second halves of the task and compared the difference in PSE values between the two halves using a paired *t* test, given that the data were normally distributed.

### Procedures and experimental design for the fMRI experiment

**Figure 2.**
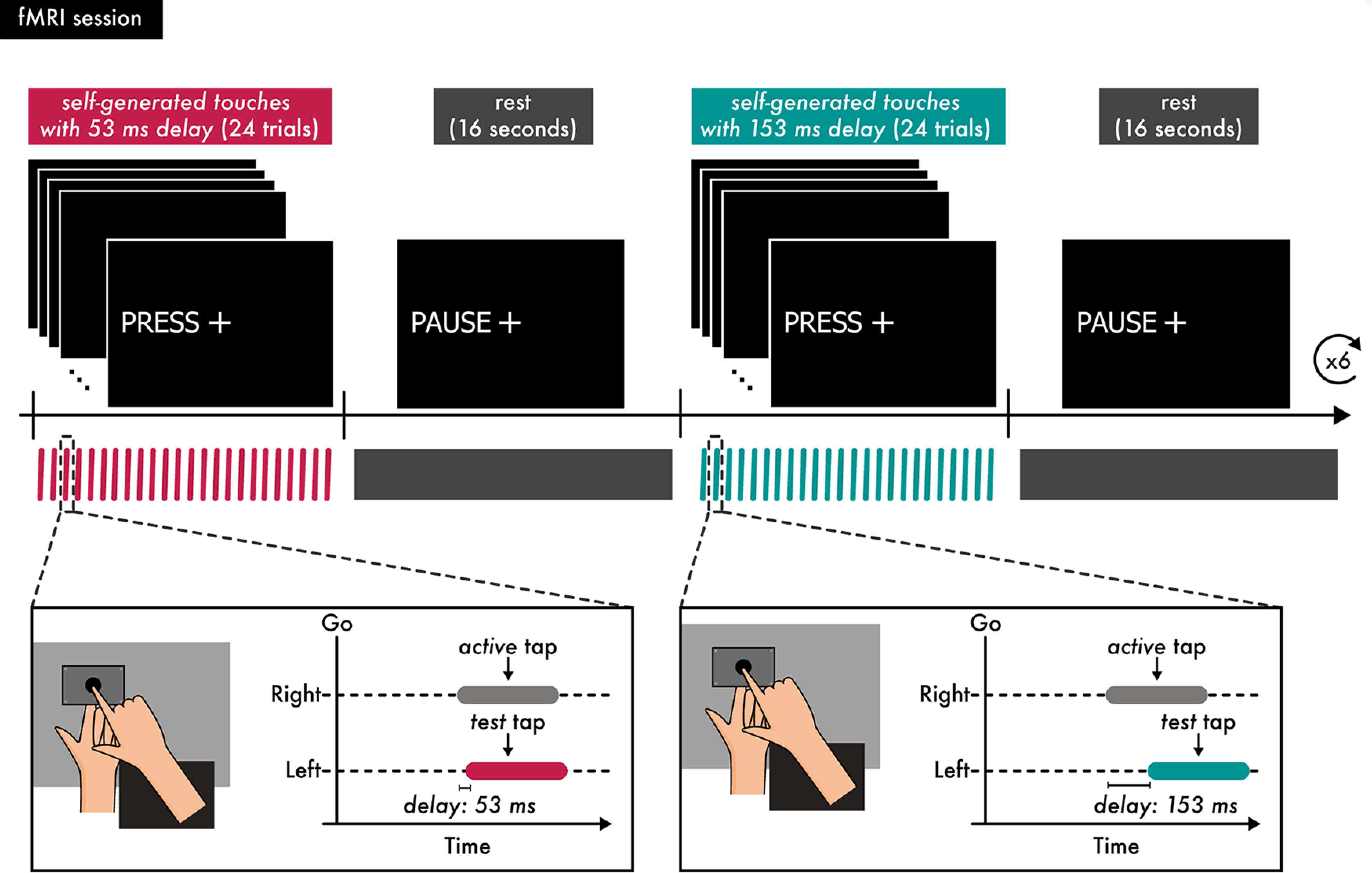
The fMRI session. The functional run was organized in blocks during which the participants produced a self-generated touch and blocks in which they remained relaxed. The run began with a block in which participants received the message PRESS on the screen; this message instructed them to tap the force sensor with their right hand (active tap) and receive the test tap on their left index finger (2 N, with a 53 ms or 153 ms delay). Participants were instructed to perform 24 such trials. In the next block, participants received the message PAUSE, which instructed them to relax both their hands for 16 s. The next block again instructed participants to produce 24 self-generated taps (with a 53 ms or 153 ms delay), followed by a rest block of 16 s. Each block of self-generated touches was repeated six times, and blocks of the different conditions were alternating. The proportions of the self-generated touch trials with the 53 ms and 153 ms delays were equal (50%).

The fMRI session always preceded the force discrimination task for practical reasons. Using the same equipment and identical to the psychophysical session, the participants were asked to tap the force sensor with their right index finger (active tap) after the auditory Go cue and received the test tap on their left index finger (2 N), with the 53 ms or 153 ms delay. Blocks including 24 such trials (with 53 ms or 153 ms) were interleaved with rest blocks of 16 s during which the subjects remained relaxed (Figure 2). We chose alternating blocks consisting of only 24 trials to avoid learning of the 153 ms delay because of repeated exposure to the delay, given our previous study (Kilteni et al., 2019) showing that >400 exposure trials are needed for participants to adapt to a 100 ms sensorimotor delay. Messages were displayed on a screen seen through a mirror attached to the head coil; these messages instructed participants regarding subsequent actions (PRESS or PAUSE). Participants were asked to fixate their gaze on the fixation cross seen on the screen and follow the messages. The participants' right arm and hand were peripherally visible. There were 12 blocks of self-generated touches (6 with the 53 ms delay and 6 with the 153 ms delay) and 12 blocks of rest, resulting in 144 self-generated touch trials with the 53 ms delay and 144 self-generated touch trials with the 153 ms delay. The condition blocks alternated, and their order was randomized among participants. On average, participants administered an active tap of (mean ± SEM) 2.084 ± 0.236 N with their right index finger and received a test tap of 1.996 ± 0.007 N on their left index finger. As in the psychophysical task, the mean duration of the active tap produced by the participants was ∼180 ms (mean ± SEM, 176.049 ± 9.416 ms), whereas the duration of the test tap produced by the setup was ∼250 ms (mean ± SEM, 241.175 ± 4.604 ms).

### Preprocessing, hypotheses, and primary statistical analysis of fMRI activations

fMRI acquisition was performed using a General Electric 3T scanner (GE750 3T) equipped with an eight-channel head coil. Gradient echo T2*-weighted EPI sequences with BOLD contrast were used as an index of brain activity. A functional image volume was composed of 42 slices (repetition time, 2000 ms; echo time, 30 ms; flip angle, 80°; slice thickness, 3 mm; slice spacing, 3.5 mm; matrix size, 76 × 76; in-plane voxel resolution, 3 mm). A total of 330 functional volumes were collected for each participant. For the anatomic localization of activations, a high-resolution structural image containing 180 slices was acquired for each participant before the acquisition of the functional volumes (repetition time, 6.404 ms; echo time, 2.808 ms; flip angle, 12°; slice thickness, 1 mm; slice spacing, 1 mm; matrix size, 256 × 256; voxel size, 1 mm × 1 mm × 1 mm).

We ran a standard preprocessing pipeline using the CONN toolbox (version 21a; Whitfield-Gabrieli and Nieto-Castanon, 2012), including realignment, unwarping, and slice-time correction. Outlier volumes were detected using the Artifact Detection Tools, employing the option for liberal thresholds (global-signal threshold of *z* = 9 and subject-motion threshold of 2 mm). Next, we simultaneously segmented the images into gray matter, white matter, and CSF and normalized them into standard Montreal Neurological Institute (MNI) space. Next, the images were spatially smoothed using an 8 mm FWHM Gaussian kernel. The structural images were also simultaneously segmented (into gray and white matter and CSF) and normalized to MNI space.

The preprocessed data were analyzed with a general linear model for each participant in Statistical Parametric Mapping 12 (SPM12; Welcome Department of Cognitive Neurology; http://www.fil.ion.ucl.ac.uk/spm). We used an event-related design with trial onsets defined as the time when the magnitude of the test tap peaked and trial durations of zero. Regressors of interest were included for each of the two conditions of interest (self-generated touch with the 53 ms and 153 ms delays). Similar to the psychophysical session, any trials in which the participants did not tap the sensor with their right index finger after the auditory cue, tapped too lightly to trigger the touch on the left index finger (active tap <0.4 N), tapped more than once, or tapped before the auditory Go cue were excluded from the regressors of interest and modeled as four individual regressors of no interest. This resulted in the exclusion of 119 trials of 8064 fMRI trials from the main regressors (1.48%). In addition, the six motion parameters and any outlier volumes were included as regressors of no interest. The trials of each condition were convolved with the canonical hemodynamic response function of SPM 12. The first-level analysis was restricted to gray matter voxels using a binary (threshold of 0.2) and smoothed mask (8 mm FWHM Gaussian kernel) of gray matter, which was based on the individual's segmented structural image (gray matter). Contrasts between the two condition regressors of interest (self-generated touch with the 153 ms delay > self-generated touch with the 53 ms delay, and self-generated touch with the 53 ms delay > self-generated touch with the 153 ms delay) were created. In the second-level analysis, random-effects group analyses were performed by entering the contrast images from each subject into a one-sample *t* test. Contrasts of interest focused on the comparisons of a self-generated touch with the 153 ms delay > a self-generated touch with the 53 ms delay, and a self-generated touch with the 53 ms delay > a self-generated touch with the 153 ms delay.

We hypothesized that the activity of the right somatosensory cortices would differ between the two self-generated touch conditions. To correct for multiple comparisons in right somatosensory areas, we performed small-volume corrections within spherical regions of interest (ROIs) of 10 mm radius, centered at peaks detected in our previous study using the same scanner, same equipment, and same tactile stimulation (2 N) applied to the same finger (left index finger; Kilteni and Ehrsson, 2020). These peaks corresponded to the right primary somatosensory cortex (S1; MNI coordinates, *x* = 50, *y* = −20, *z* = 60) and the right secondary somatosensory cortex (rSII; MNI coordinates, *x* = 46, *y* = −14, *z* = 16). To correct for multiple comparisons within the cerebellum, we used anatomic masks created with the Anatomy Toolbox (Eickhoff et al., 2005), including the hemispheres of the right and left lobules V, VI, and VIII, given the involvement of these cerebellar regions in the sensorimotor cerebellar body representation (Grodd et al., 2001; Diedrichsen et al., 2005; Stoodley and Schmahmann, 2009; O'Reilly et al., 2010; Buckner et al., 2011; Bostan et al., 2013; Guell et al., 2018; King et al., 2018). To directly compare our results with those from Blakemore et al.'s (2001) study using positron emission tomography (PET), we also included a mask containing the right lobule VIIa Crus I, given that the authors reported peaks in both lobules VI and VIIa Crus I. In addition to these hypothesized regions, we report analyses at the whole-brain level.

For each peak activation, the coordinates in MNI space, the *z* value, and the *p* value are reported. We denote that a peak survived a threshold of *p* < 0.05 after correction for multiple comparisons at the whole-brain or small-volume level by adding the term FWE corrected after the *p* value.

### Statistical analysis of the relationship between fMRI activations (self-generated touch with the 153 ms delay > self-generated touch with the 53 ms delay) and the psychophysical results

We evaluated the relationship between the perceptual differences in force discrimination revealed by the psychophysical task and the effects revealed by our fMRI univariate analysis. To do so, we extracted the signal from the contrast estimates of each condition against zero (self-generated touch with the 53 ms delay >0 and self-generated touch with the 153 ms delay >0) using the MarsBaR toolbox (Brett et al., 2002) at the peaks where the activity significantly differed between the two conditions (*p* < 0.05, FWE corrected). We then performed a standard correlation analysis of the signal difference between the two conditions at the significant peaks with the difference in the PSE values extracted from the psychophysical task (*PSE*_53ms_ – *PSE*_153ms_).

### fMRI functional connectivity: preprocessing, hypotheses, and statistical analysis

For the functional connectivity analysis, data were further denoised using the component-based noise correction method (CompCor) as implemented in the CONN toolbox. Five principal components from white matter, 5 principal components from CFS, 12 principal realignment components (6 plus first-order derivatives) and scrubbing parameters, together with 2 principal components per condition (the time series and its first derivative), were extracted and treated as confounds. A high-pass filter (cutoff frequency = 0.008 Hz) was applied, and the data were linearly detrended.

We previously showed that the degree of functional connectivity between the cerebellum and the somatosensory areas is linearly and positively related to the degree to which participants perceptually attenuate their self-generated touches (Kilteni and Ehrsson, 2020). Therefore, we hypothesized that the right somatosensory cortices would decrease their connectivity with the cerebellum when a temporal perturbation is present as a function of the participants' perception. To test this hypothesis, we conducted a seed-to-voxel analysis in the form of generalized psychophysiological interactions (gPPI; McLaren et al., 2012) using the denoised data. Right somatosensory seeds of interest were defined as spheres with an 8 mm radius around the two somatosensory peaks (right S1 and right SII, *p* < 0.05, FWE corrected) revealed by the activation analysis (self-generated touch with the 153 ms delay > self-generated touch with the 53 ms delay) of the present study. At the group level, the contrasts of interest focused on the effect of delay (self-generated touch with the 153 ms delay > self-generated touch with the 53 ms delay), and we identified both increases and decreases in the functional connectivity of the seeds. To specifically identify any connectivity changes in the somatosensory seeds that scaled with the participants' perception, we used the PSE difference from the psychophysical task as a second-level covariate (*PSE*_153ms_ – *PSE*_53ms_).

Given that the supplementary motor area may provide the efference copy to predict and attenuate self-generated somatosensory activity (Haggard and Whitford, 2004), but also use information related to discrepancies between the predicted and actual feedback to update the motor plan (Welniarz et al., 2021), we further hypothesized that there would be differences in connectivity of the left SMA between conditions. Specifically, we anticipated that the left SMA would increase its connectivity with the left CB in the presence of temporal perturbations because of the feedback signal, indicating the temporal discrepancy between the predicted and actual touch on the left index finger. At the same time, the left SMA should decrease its connectivity with the right somatosensory cortices during temporal perturbations, indicating reduced attenuation of the somatosensory reafference on the left hand, similar to our hypothesis about the cerebellum. To evaluate the left SMA connectivity, we placed a seed of interest (8-mm-radius sphere) at the peak corresponding to the left supplementary motor area that showed significant activation in both condition contrasts against zero (*p* < 0.05, FWE corrected). Because we did not have a hypothesis regarding whether these theorized effects would be mediated by the participants' somatosensory perception, we performed two connectivity analyses, with and without the participants' perceptual changes (*PSE*_153ms_ – *PSE*_53ms_) as a covariate.

Statistical maps were assessed with corrections for multiple comparisons using either anatomic masks or peaks from our previous study (Kilteni and Ehrsson, 2020). When using the somatosensory seeds (the right S1 or right SII), we corrected for multiple comparisons within the cerebellum by performing small-volume corrections within anatomic masks (right and left lobules V, VI, and VIII), identical to our univariate analysis. To correct for the left SMA, we used a spherical ROI (10 mm radius) around the left supplementary motor area peak detected in our previous study (MNI coordinates, *x* = −6, *y* = −8, *z* = 54; Kilteni and Ehrsson, 2020). When using the left SMA as seed, we corrected for multiple comparisons within the somatosensory areas by performing small-volume corrections within the spherical ROIs (10 mm radius) centered at the two peaks (the right S1 and right SII) detected in our previous study (Kilteni and Ehrsson, 2020), identical to our univariate analysis. Corrections for multiple comparisons within the cerebellum were performed using the above mentioned masks.

### Complementary post hoc fMRI analyses

In a subsequent analysis, we explored the potential influence of small variations in the magnitude of the self-generated force of active taps on the BOLD signal. Previous studies have established that larger muscular forces can produce increased BOLD signals in the primary motor cortex, posterior supplementary motor area, cerebellum, and secondary somatosensory cortex (Dettmers et al., 1995; Ehrsson et al., 2001), although these studies used much greater variations in force than small variations expected in the present study. We followed the same modeling approach described above, but we also included the magnitude of the active tap on each trial as a parametric modulator for all the trials of the two conditions of interest. The two contrasts of interest focused on the overall modulation of the active taps across both conditions (*pmod*_153ms_ + *pmod*_53ms_ > 0) and the effect of delay (self-generated touch with the 153 ms delay > self-generated touch with the 53 ms delay). We expected that the left motor cortex (M1) and the right CB would exhibit increased activity as a function of the magnitude of the active tap; that is, stronger forces generated by the right hand would elicit greater motor activity in the left hemisphere and greater cerebellar activity in the right hemisphere. To test these hypotheses, we performed small-volume corrections within a spherical ROI centered at the left primary motor cortex (MNI coordinates, *x* = −38, *y* = −12, *z* = 52) detected in our previous study (Kilteni and Ehrsson, 2020) and within the above mentioned anatomic cerebellar masks. Then, we conducted an additional control analysis for the condition-specific contrasts (self-generated touch with the 153 ms delay > self-generated touch with the 53 ms delay, and self-generated touch with 53 ms delay > self-generated touch with the 153 ms delay) by including the force parametric modulator in the model and regressing out force-related signal variation in the data.

Similar to our control analysis in the psychophysics task, we performed a control analysis to test for significant learning effects because of repeated exposure to the 153 ms delay. As mentioned above, significant learning of a 100 ms delay requires >400 exposure trials (Kilteni et al., 2019). To avoid learning of the 153 ms in the present study and identify genuine differences between the self-generated touch with the 53 ms delay and the self-generated touch with the 153 ms delay self-generated touch conditions, we thus designed the run to include only 24 trials in each block, with the type of blocks alternating. If there was adaptation, this may have reduced the effect of the brief temporal perturbations on the BOLD signal, especially toward the end of the fMRI run. To confirm that no such delay adaptation occurred, we modeled the trials of each condition separately for each block (first, second, third, fourth, fifth, and sixth), resulting in 12 different regressors. We then created contrasts of each condition against zero for the first and the last block (e.g., self-generated touch with 53 ms *delay*_block1_ > 0, self-generated touch with 153 ms *delay*_block1_ > 0, self-generated touch with 53 ms *delay*_block6_ > 0, self-generated touch with 153 ms *delay*_block6_ > 0), and we extracted the activity from each contrast at the peak voxels revealed by the univariate analysis using the MarsBaR Toolbox (Brett et al., 2002). We then performed a paired *t* test on the difference of the two conditions between the first and the last block, given that the data were normally distributed.

### Anatomical localizations

The activity was anatomically localized based on macroanatomical landmarks (sulci and gyri) using the terminology from the Duvernoy (1999) brain atlas as well as on the Anatomy Toolbox (Eickhoff et al., 2005, 2007). In addition, we used the SUIT toolbox for anatomic localization based on a probabilistic atlas of the cerebellum (Diedrichsen et al., 2009).

## Results

### Temporal perturbations disrupt the perceptual attenuation of somatosensory reafference

**Figure 3.**
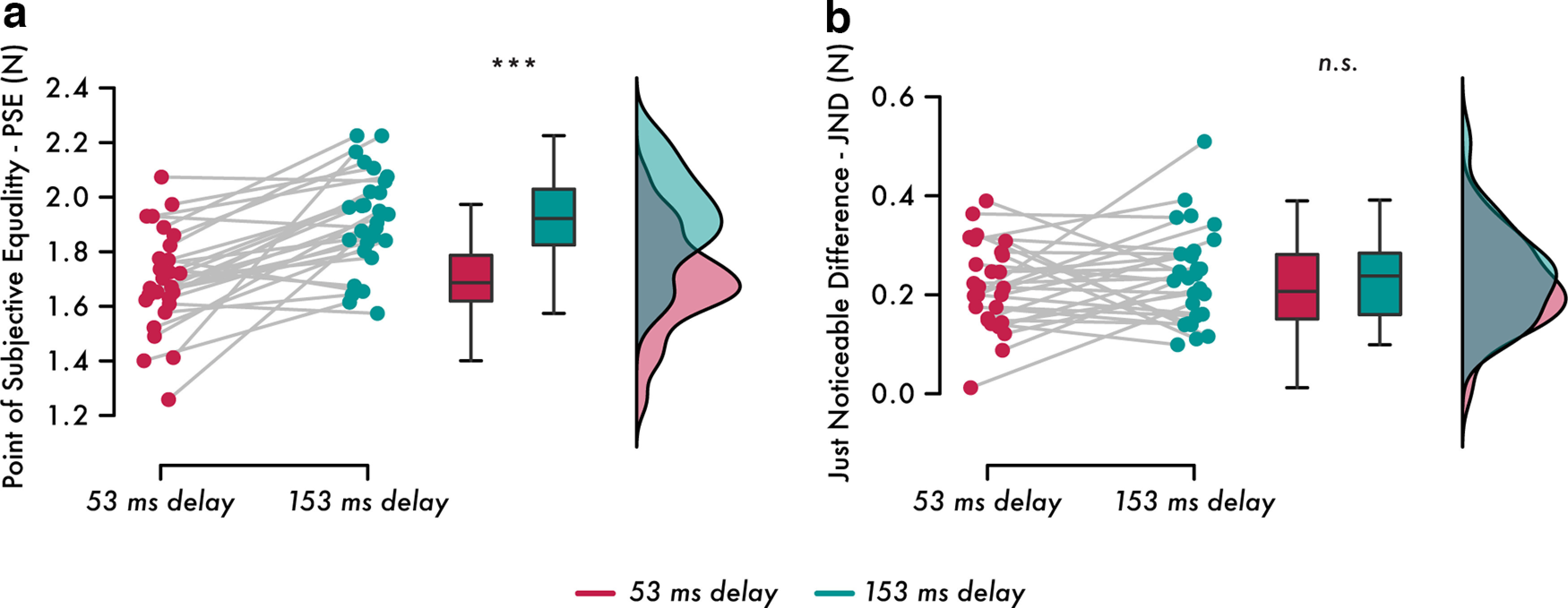
Results of the psychophysics task. ***a***, Individual PSE values and line plots illustrating the decreased PSE in the self-generated touch with the 53 ms delay compared with the self-generated touch with the 153 ms delay condition (*p* < 0.001). Box plots and raincloud plots illustrate the group effects (Extended Data Figs. 3-1, 3-2). ***b***, Individual JND values and line plots illustrate the nonsignificant JND changes between the self-generated touch with the 53 ms delay and self-generated touch with the 153 ms delay conditions. Box plots and raincloud plots illustrate the group effects. ***p < 0.001, n.s. not significant.

For all participants and all conditions, the logistic models had very good fits, with McFadden's *R*^2^ values ranging between 0.409 and 0.945 (Extended Data Fig. 3-1). The self-generated touch with the 53 ms delay condition induced a significant decrease in the PSE (i.e., attenuation) compared with the self-generated touch with the 153 ms delay condition (*n* = 28, *t*_(27)_ = −5.726, *p* < 0.001, Cohen's *d* = −1.082; CI^95^, −0.297, −0.140) despite having identical intensities (i.e., 2 N; Fig. 3*a*). This decrease in PSE values was observed for 23 of 28 participants (82.1%); that is, 23 participants had a lower PSE in the self-generated touch with the 53 ms delay condition than in the self-generated touch with the 153 ms delay condition. Together, these findings extend previous results (Blakemore et al., 1999; Bays et al., 2005; Kilteni et al., 2019, 2021) showing that self-generated touches feel stronger when delivered with a 153 ms temporal perturbation than an identical self-generated touch delivered close to the predicted time of contact between the two fingers (i.e., 53 ms). Moreover, there was no difference in the force discrimination capacity (JND) between the two conditions (*n* = 28, *t*_(27)_ = −1.048, *p* = 0.304, Cohen's *d* = −0.198; CI^95^, −0.068, 0.022; Fig. 3*b*). Specifically, 14 participants (50%) had increased JND values, and 14 participants (50%) had decreased JND values between conditions. A Bayesian analysis also supported the absence of a JND difference (*BF*_01_ = 3.033). Together, the psychophysical results indicate that the attenuation of somatosensory reafference observed when the touch is delivered close to its expected time (i.e., with 53 ms delay; PSE) becomes disrupted when the same touch is delivered with a 153 ms delay but without influencing somatosensory precision (JND; Asimakidou et al., 2022; Kilteni and Ehrsson, 2022). Thus, overall, the psychophysical results verified that our behavioral paradigm worked as expected in the scanner environment.

10.1523/JNEUROSCI.1743-22.2023.f3-1 Figure 3-1 Individual plots of the psychophysical session. The marker size is proportional to the number of repetitions at that stimulus level. For all participants and conditions, the fitted model resulted in a McFadden’s *R*^2^ value ranging between 0.409 and 0.945. Download Figure 3-1, TIF file.

10.1523/JNEUROSCI.1743-22.2023.f3-2 Figure 3-2 Absence of learning effects of the 153 ms delay during the psychophysical session. Individual differences and line plots illustrating the difference in the PSE values of the two conditions (self-generated touch with the 153 ms delay – self-generated touch with the 53 ms delay self-generated touch) between the first and the second halves of the psychophysical task. Box plots and raincloud plots illustrate the group effects. There were no learning effects, as strongly supported by a Bayesian analysis. Download Figure 3-2, TIF file.

### No changes in psychophysical responses evoked by temporal perturbations over time

As we expected, and in agreement with our previous results (Kilteni et al., 2019), we found no evidence that the temporal perturbation of 153 ms was learned (i.e., no differences in performance on the first and the second halves of the psychophysical task; *n* = 28, *t*_(27)_ = 0.418, *p* = 0.679, Cohen's *d* = 0.079; CI^95^, −0.099, 0.149). A Bayesian analysis also provided evidence of the absence of a learning effect (*BF*_01_ = 4.602; Extended Data Fig. 3-2).

### Temporal perturbations disrupt the attenuation of somatosensory reafference in the right primary and secondary somatosensory cortices and the right cerebellum

**Figure 4.**
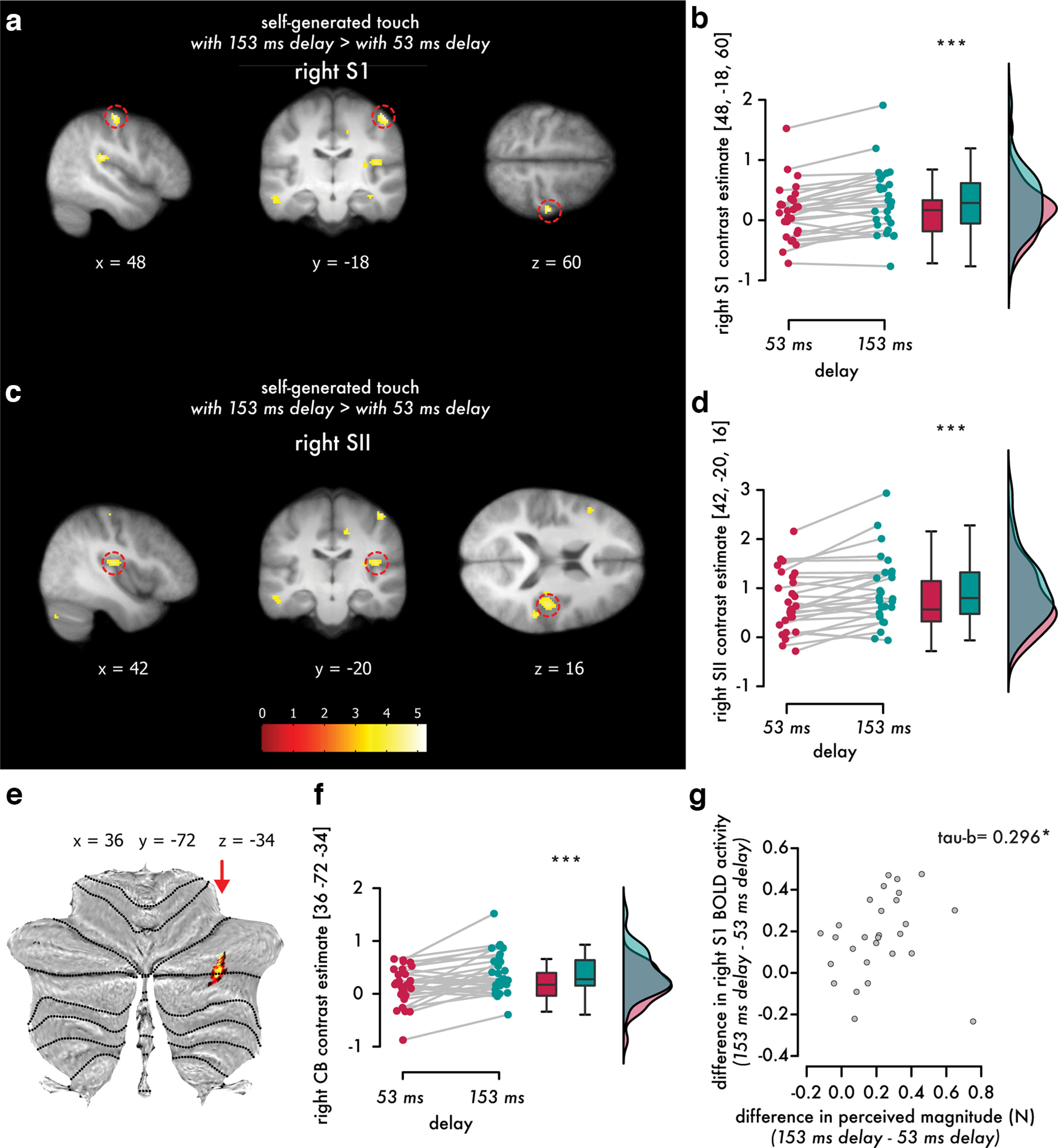
Somatosensory and cerebellar activations elicited during the self-generated touch with the 153 ms delay condition compared with the self-generated touch with the 53 ms delay condition. ***a***, ***c***, Sagittal (left), coronal (middle), and axial (right) views of significant peaks of activation (*p* < 0.05, FWE corrected) located in the right primary (postcentral gyrus) and secondary somatosensory cortex (parietal operculum). The activation maps are rendered on the mean structural image across all 28 participants and are displayed at an uncorrected threshold of *p* < 0.001. Red circles indicate the main significant peaks. The color bar indicates the values of the *t* statistic (Exended Data Figs. 4-1, 4-2, 4-3; Extended Data Tables 4-1, 4-2, 4-3, 4-4, 4-5, 4-6). ***e***, The cerebellar activations are rendered on a flat representation of the human cerebellum (Diedrichsen and Zotow, 2015) at an uncorrected threshold of *p* < 0.001. The red arrow indicates the significant peak within lobule VIIa Crus I (*p* < 0.05, FWE corrected). ***b***, ***d***, ***f***, Individual contrast estimates and line plots illustrating the increase in the activation of the right S1 (***b***), right SII (***d***), and right CB (***f***) in the self-generated touch with 153 ms delay compared with the self-generated touch with 53 ms delay conditions (Exended Data Fig. 4-4). All data were corrected for multiple comparisons (*p* < 0.05, FWE corrected). ***g***, Scatter plot showing the statistically significant and positive relationship between the difference in the perceived magnitude between the two conditions (i.e., difference in PSE values between the self-generated touch with the 153 ms delay and self-generated touch with the 53 ms delay conditions) and the difference in the BOLD activity of the right S1 between the self-generated touch with the 153 ms delay and the self-generated touch with the 53 ms delay conditions. (***p < 0.001,*p < 0.05).

Compared with the baseline (rest blocks), both self-generated touch conditions elicited significant neural activity (*p* < 0.05, FWE corrected) in the contralateral premotor and motor cortices, supplementary motor area, and bilateral somatosensory and cerebellar areas, as expected (Extended Data Tables 4-1, 4-2; Extended Data Fig. 4-1). Importantly, when directly comparing the two conditions, the self-generated touch with the 153 ms delay condition elicited increased activity in the right S1 (postcentral gyrus; MNI coordinates, *x* = 48, *y* = −18, *z* = 60, *p* = 0.002, FWE corrected; *x* = 50, *y* = −16, *z* = 56, *p* = 0.002, FWE corrected), and the right SII (parietal operculum; MNI coordinates, *x* = 42, *y* = −20, *z* = 16, *p* = 0.006, FWE corrected) compared with the self-generated touch with the 53 ms delay condition (Fig. 4*a–d*, Extended Data Table 4-3). Moreover, the self-generated touch with the 153 ms delay condition elicited increased activity in the right CB (MNI coordinates, *x* = 36, *y* = −72, *z* = −34, *p* = 0.049, FWE corrected) compared with the self-generated touch with the 53 ms delay condition (Fig. 4*e*,*f*) in lobule VIIa Crus I. No significant differences were observed in the hemispheres of other cerebellar lobules. The opposite contrast (self-generated touch with the 53 ms delay > self-generated touch with the 153 ms delay) mainly revealed activity in the right middle frontal gyrus that did not survive corrections for multiple comparisons and will therefore not be considered further (Extended Data Fig. 4-2, Extended Data Table 4-4). Together, these findings show that a self-generated touch elicits stronger somatosensory and cerebellar activity when delivered perturbed by 153 ms compared with an identical self-generated touch delivered close to its expected timing (i.e., 53 ms delay). In other words, the neural attenuation of somatosensory reafference in the somatosensory and cerebellar cortices observed when the touch is delivered close to its expected time (i.e., 53 ms) becomes disrupted when the same touch is delivered with a temporal perturbation (i.e., 153 ms).

10.1523/JNEUROSCI.1743-22.2023.f4-1 Figure 4-1 Activations during the self-generated touch with the 53 ms delay and self-generated touch with the 153 ms delay conditions. ***a***, ***b***, Activations reflect greater effects of the self-generated touch with the 53 ms delay condition than the rest condition. ***c***, ***d***, Activations reflect greater effects during the self-generated touch with the 153 ms delay condition than the rest condition. In both contrasts, auditory areas were also activated because the participants heard auditory GO cues to produce the self-generated touches. The activations were rendered on the standard single-subject 3D volume provided with SPM (***a***, ***c***). Cerebellar activations were overlaid onto a cerebellar flat map (***b***, ***d***). All activation maps (***a–d***) are displayed at a threshold of p < 0.05, FWE corrected. Download Figure 4-1, TIF file.

10.1523/JNEUROSCI.1743-22.2023.f4-2 Figure 4-2 Activations elicited during the self-generated touch with the 53 ms delay compared with the self-generated touch with the 153 ms delay conditions. ***a***, Activations reflect greater effects during the self-generated touch with the 53 ms delay compared with the self-generated touch with the 153 ms delay in the right middle frontal gyrus that did not survive corrections for multiple comparisons. The activations are rendered on the mean structural image across all participants. All activation maps are displayed at a threshold of *p* < 0.001 uncorrected (Exended Data Table 4-4). ***b***, Individual contrast estimates and line plots illustrating the increase in the activation of the middle frontal gyrus in the self-generated touch with the 53 ms delay compared with the self-generated touch with the 153 ms delay conditions (*p* < 0.001). Download Figure 4-2, TIF file.

10.1523/JNEUROSCI.1743-22.2023.f4-3 Figure 4-3 ***a***, ***b***, Sensorimotor (***a***) and cerebellar (***b***) areas whose BOLD activity was significantly and linearly modulated by the forces participants exerted with their right index finger (active taps). The activity of the left motor cortex was significantly modulated by the strength of the active taps (***a***). The cluster extends to the left primary somatosensory cortex. The red circle indicates the significant peak. The activations were rendered on the mean structural image across all participants at an uncorrected threshold of *p* < 0.001. Multiple peaks in the right and left cerebellum (***b***) were significantly modulated by the taps of the participants’ right hand (lobules V, VI). Arrows denote the significant peaks. The cerebellar activations were rendered on the cerebellar flat map at an uncorrected threshold of *p* < 0.001. Download Figure 4-3, TIF file.

10.1523/JNEUROSCI.1743-22.2023.f4-4 Figure 4-4 Absence of learning effects of the 153 ms delay during the fMRI run. ***a–c***, Individual differences and line plots illustrating the difference in the extracted activity for contrast estimates between the two conditions (self-generated touch with the 153 ms delay – self-generated touch with the 53 ms delay) between the first and the last block of the fMRI run in the (***a***) right primary somatosensory cortex (rS1), (***b***) secondary somatosensory cortex (rSII), and (***c***) the right cerebellum (rCB). Box plots and raincloud plots illustrate the group effects. There were no learning effects, as strongly supported by a Bayesian analysis. Download Figure 4-4, TIF file.

10.1523/JNEUROSCI.1743-22.2023.t4-1 Table 4-1 Activation peaks for the self-generated touch with the 53 ms delay condition. Peaks reflect greater effects during self-generated touch with the 53 ms delay compared with rest (self-generated touch with the 53 ms delay >0). Only the peaks that survived the FWE correction (*p* ***<*** 0.05) belonging to clusters with a size > four voxels are reported for spatial restrictions. Download Table 4-1, DOCX file.

10.1523/JNEUROSCI.1743-22.2023.t4-2 Table 4-2 Activation peaks for the self-generated touch with the 153 ms delay condition. Peaks reflect greater effects during self-generated touch with the 153 ms delay compared with rest (self-generated touch with the 153 ms delay >0). Only the peaks that survived the FWE correction (*p* < 0.05) belonging to clusters with a size > four voxels are reported for spatial restrictions. Download Table 4-2, DOCX file.

10.1523/JNEUROSCI.1743-22.2023.t4-3 Table 4-3 Activations greater during the self-generated touch with the 153 ms delay than the self-generated touch with the 53 ms delay conditions. Peaks reflect greater effects of the self-generated touch with the 153 ms delay compared with the self-generated touch with the 53 ms delay conditions. Download Table 4-3, DOCX file.

10.1523/JNEUROSCI.1743-22.2023.t4-4 Table 4-4 Activations greater during the self-generated touch with the 53 ms delay than the self-generated touch with the 153 ms delay conditions. Peaks reflect greater effects of the self-generated touch with the 53 ms delay compared with self-generated touch with the 153 ms delay conditions. Download Table 4-4, DOCX file.

10.1523/JNEUROSCI.1743-22.2023.t4-5 Table 4-5 Areas whose activity was parametrically modulated by the strength of the right-hand active taps across the self-generated touch with the 53 ms and the 153 ms delay conditions. Download Table 4-5, DOCX file.

10.1523/JNEUROSCI.1743-22.2023.t4-6 Table 4-6 Activations greater during the self-generated touch with the 153 ms delay than the self-generated touch with the 53 ms delay conditions, with active taps as parametric modulator. Peaks reflect greater effects of the self-generated touch with the 153 ms delay compared with the self-generated touch with the 53 ms delay conditions. Download Table 4-6, DOCX file.

Including the forces generated by the right index finger (active taps) as a parametric modulator of each trial and examining its effect on BOLD activity revealed significant activity in the left motor cortex (precentral gyrus) expanding to the left primary somatosensory cortex (postcentral gyrus) and bilateral cerebellum (lobules V and VI; Extended Data Table 4-5). That is, the intensity of the active taps the participants pressed with their right index finger parametrically modulated the activity in contralateral sensorimotor and bilateral cerebellar cortices. No modulation of the right somatosensory or motor cortex was detected (even at *p* < 0.005, uncorrected); i.e., the effects produced by the force production of the right hand and observed in the left hemisphere were anatomically distinct from the somatosensory effects in the right somatosensory cortex contralateral to the passive left index finger receiving the tactile stimulation (as reported above). Notably, when including the parametric modulator in the main analysis contrasting the self-generated touch with the 153 ms delay and the self-generated touch with the 53 ms delay conditions, we found the same somatosensory effects in the right S1 and right SII as those in the main analysis reported above (Extended Data Table 4-6). This rules out the possibility that small variations across trials in muscular contractions, produced force, or the associated somatosensory feedback from the right index finger explain our main findings.

### Disruption of perceptual attenuation because of temporal perturbations predicts the disruption of neural attenuation in primary somatosensory responses

We then investigated whether the increase in PSE values because of temporal perturbations (Fig. 3*a*) was related to the increased responses of the right S1, right SII, and right cerebellum (Fig. 4*a*,*c*,*e*). We calculated the difference in PSE values between the self-generated touch with the 153 ms delay and the self-generated touch with the 53 ms delay conditions and the difference in contrast estimates for the activation peaks in the right S1 (MNI coordinates, *x* = 48, *y* = −18, *z* = 60), right SII (MNI coordinates, *x* = 42, *y* = −20, *z* = 16) and right cerebellum (MNI coordinates, *x* = 36, *y* = −72, *z* = −34) between the self-generated touch with the 153 ms delay and the self-generated touch with the 53 ms delay conditions. The increase in PSE values was significantly and positively correlated with the increase in the responses of the right S1 (*n* = 28, Kendall's *tau-b* = 0.296, *p* = 0.027; Fig. 4*g*). No such relationship was found in the right SII (*n* = 28, Pearson's ρ = −0.083, *p* = 0.674) or in the right cerebellum (*n* = 28, Pearson's ρ = 0.1808, *p* = 0.2604). This suggests that the disruption of attenuation in the right S1 because of temporal perturbations reflects the disruption of attenuation at the perceptual level because of the same temporal perturbations.

### Neural responses evoked by temporal perturbations do not change over time

As we expected, and in agreement with our previous results and with the psychophysical results reported above, we found no evidence of learning (i.e., no difference) between the first and the last scanning block in the right S1 (*n* = 28, *t*_(27)_ = 0.955, *p* = 0.348, Cohen's *d* = 0.181; CI^95^, −0.323, 0.887), right SII (*n* = 28, *t*_(27)_ = 0.670, *p* = 0.509, Cohen's *d* = 0.127; CI^95^, −0.486, 0.956), or right CB (*n* = 28, *t*_(27)_ = 0.466, *p* = 0.645, Cohen's *d* = 0.088; CI^95^, −0.469, 0.744). A Bayesian analysis provided evidence of the absence of a learning effect (right S1, *BF*_01_ = 3.293; right SII, *BF*_01_ = 4.061, right CB, *BF*_01_ = 4.513; Extended Data Fig. 4-4).

### Temporal perturbations decrease the functional connectivity of the right primary somatosensory cortex with the left supplementary motor area, the bilateral cerebellum, and the left secondary somatosensory cortex, proportional to the reduction in somatosensory perception

**Figure 5.**
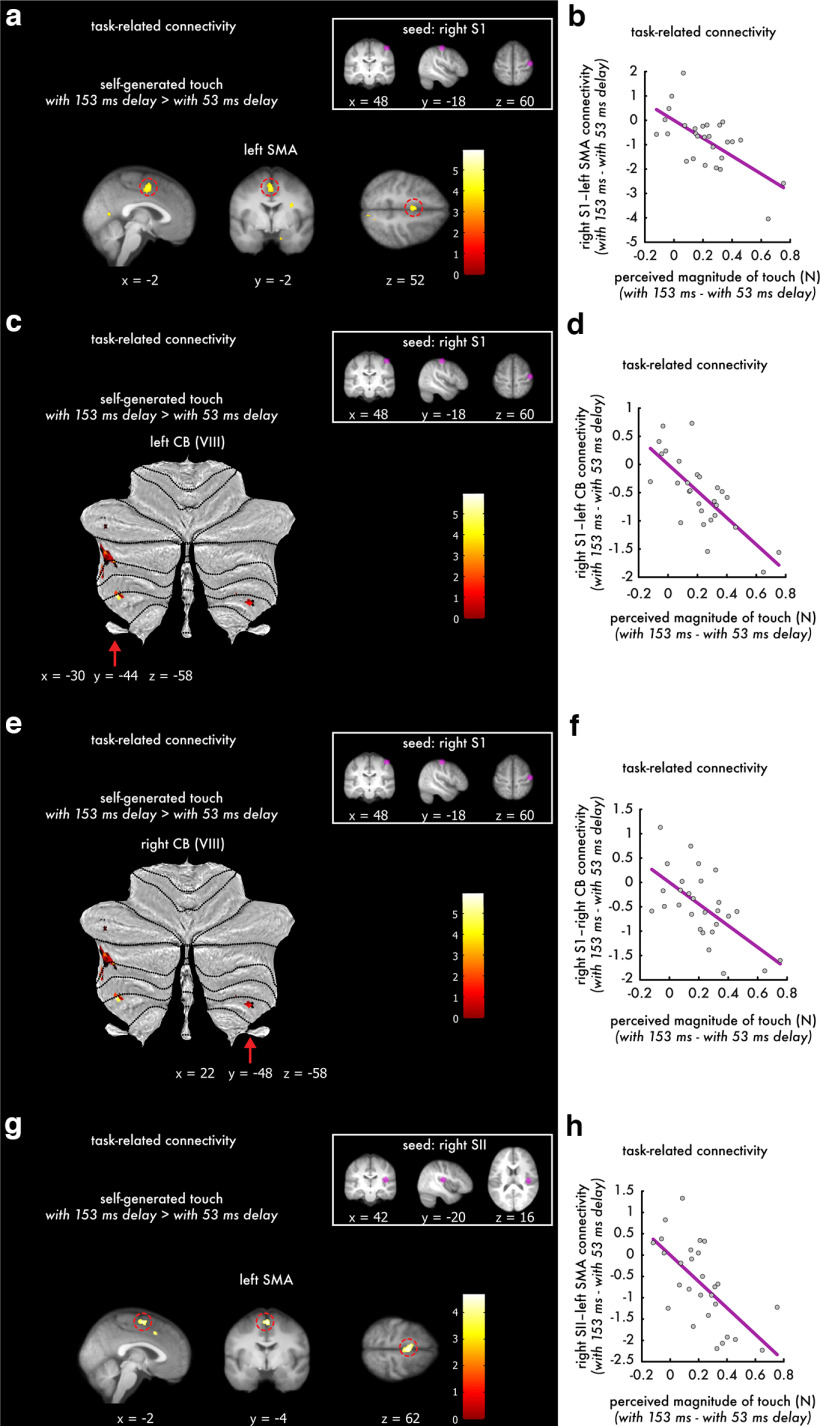
***a–h***, Functional connectivity results when setting the right S1 (***a–f***) or right SII (***g***, ***h***) as seeds showing decreased functional connectivity with the left SMA and bilateral cerebellum as a function of psychophysical somatosensory perception. ***a***, Sagittal (left), coronal (middle), and axial (right) views of the significant peak in the left SMA (*p* < 0.05, FWE corrected) that decreased connectivity with the right S1 (seed) in the gPPI. Activations have been rendered on the mean structural image across all participants at a threshold of *p* < 0.001 uncorrected, and the red circles indicate the significant peaks. Cerebellar flat maps showing left (***c***) and right (***e***) cerebellar areas (VIII) with decreased connectivity to the right S1. The cerebellar activations have been rendered on the cerebellar flat map at a threshold of *p* < 0.001 uncorrected, and red arrows indicate the locations of the significant peaks (*p* < 0.05, FWE-corrected). ***g***, Sagittal (left), coronal (middle), and axial (right) views of the significant peak in the left SMA (*p* < 0.05, FWE corrected) that decreased connectivity with the right SII (seed). Activations have been rendered on the mean structural image across all participants at a threshold of *p* < 0.001 uncorrected, and the red circles indicate the significant peaks (*p* < 0.05, FWE corrected). ***b***, ***d***, ***f***, ***h***, Scatter plots showing the relationship of connectivity decreases between the corresponding seed and the significant peaks **(*a***, ***c***, ***e***, ***g*)** with the participants' PSE differences extracted from the force discrimination task (Exended Data Table 5-1). Each marker represents one participant. The color bars indicate the values of the *t* statistic.

We expected that the disruption in the predictive processing of somatosensory reafference because of the temporal perturbations would disrupt the connectivity of the right somatosensory cortices with brain areas involved in predicting sensory consequences of the movement (i.e., the SMA and cerebellum). To test this, we performed a seed-to-voxel gPPI analysis of functional connectivity setting the right S1 or right SII as seeds and including the participants' PSE differences from the psychophysical task (Fig. 3*a*) as a covariate. This allowed us to isolate somatosensory functional connectivity increases or decreases that scaled linearly with perceptual changes in participants' somatosensory perception. We found that the right S1 showed significant decreases in connectivity with the left SMA (MNI coordinates, *x* = −2, *y* = −2, *z* = 52, *p* < 0.01, FWE corrected; Fig. 5*a*,*b*), the left cerebellar lobule VIII (MNI coordinates, *x* = −30, *y* = −44, *z* = −58, *p* = 0.001, FWE corrected; MNI coordinates, *x* = −16, *y* = −62, *z* = −60, *p* = 0.043, FWE corrected; Fig. 5*c*,*d*), the right cerebellar lobule VIII (MNI coordinates, *x* = 22, *y* = −48, *z* = −58, *p* = 0.029, FWE corrected; MNI coordinates, *x* = 20, *y* = −62, *z* = −60, *p* = 0.049, FWE corrected; Fig. 5*e*,*f*), and the left secondary somatosensory cortex (MNI coordinates, *x* = 46, *y* = −16, *z* = 24, *p* = 0.023, FWE corrected) during the self-generated touch with the 153 ms delay compared with the self-generated touch with the 53 ms delay condition (Extended Data Table 5-1). Similarly, the right SII showed a significant decrease in connectivity with the left SMA (MNI coordinates, *x* = −2, *y* = −4, *z* = 62, *p* = 0.011, FWE corrected; Fig. 5*g*,*h*). In contrast, there were no significant connectivity increases with the right S1 or SII as seeds. Together, these results indicate that the stronger the disruption of the somatosensory reafference because of the temporal perturbation at the perceptual level (difference in PSE values), the weaker the connectivity of the right S1 with the left SMA, the bilateral cerebellar lobules VIII, and the right SII, and the weaker the connectivity of the right SII with the left SMA.

10.1523/JNEUROSCI.1743-22.2023.t5-1 Table 5-1 Peaks with decreased connectivity with the left primary somatosensory cortex during the temporal perturbation as a function of the PSE difference between the self-generated touch with the 153 ms delay and self-generated touch with the 53 ms delay conditions. Peaks reflect reduced connectivity with the left primary somatosensory cortex in the self-generated touch with the 153 ms delay compared with the self-generated touch with the 53 ms delay conditions and covaried with participant perception. Only the peaks that belonged to clusters with a size greater than four voxels are reported for spatial restrictions. Download Table 5-1, DOCX file.

### Temporal perturbations increase the functional connectivity of the left supplementary motor area with the left cerebellum

**Figure 6.**
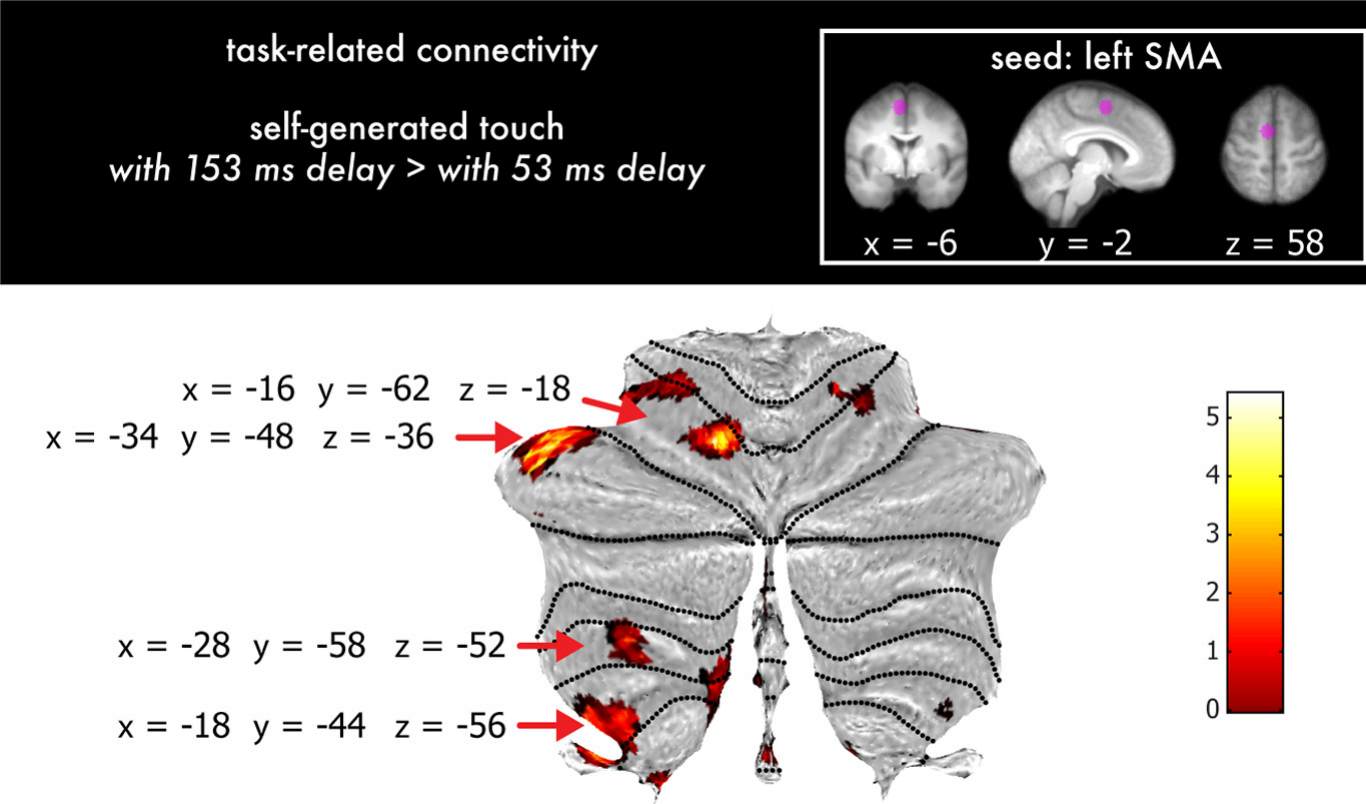
Functional connectivity results showing increased connectivity of the left SMA (seed) with the cerebellum during temporal perturbations. A gPPI revealed multiple cerebellar peaks that increased connectivity with the left supplementary motor area during temporal perturbations (self-generated touch with the 153 ms delay > self-generated touch with the 53 ms delay; Exended Data Tables 6-1, 6-2). The cerebellar activations have been rendered on the cerebellar flat map at a threshold of *p* < 0.001 uncorrected, and red arrows indicate the location of the significant peaks within lobules VI, VI/VIIa, VIIIa, and VIIIb. The color bar indicates the values of the *t* statistic.

Finally, we hypothesized that the temporal perturbations would increase the connectivity of areas involved in motor planning (i.e., the SMA) with areas involved in processing the temporal discrepancy (i.e., the cerebellum). Such connectivity changes could reflect processing of the temporal error between the predicted and actual somatosensory input to update the motor plan if needed. A seed-to-voxel functional connectivity analysis (gPPI) setting the left SMA as the seed region confirmed this hypothesis; there were significant increases in connectivity of the left SMA with the left cerebellum (lobules VI, VI/Crus I, VIIIa, and VIIIb; Fig. 6; Extended Data Table 6-1) during the self-generated touch with the 153 ms delay compared with the self-generated touch with the 53 ms delay condition (*p* < 0.05, FWE corrected). The effects did not covary with participants' perception, and no effects were found for the opposite contrast (i.e., self-generated touch with the 53 ms delay > self-generated touch with the 153 ms delay; Extended Data Table 6-2).

10.1523/JNEUROSCI.1743-22.2023.t6-1 Table 6-1 Peaks with increased connectivity with the left supplementary motor area during temporal perturbation. Peaks reflect greater connectivity with the left supplementary motor area in the self-generated touch with the 153 ms delay compared with the self-generated touch with the 53 ms delay conditions. Only the peaks that belonged to clusters with a size greater than four voxels are reported for spatial restrictions. Download Table 6-1, DOCX file.

10.1523/JNEUROSCI.1743-22.2023.t6-2 Table 6-2 Peaks with decreased connectivity with the left supplementary motor area during temporal perturbations. Peaks reflect lower connectivity with the left supplementary motor area for the self-generated touch with the 153 ms delay compared with the self-generated touch with the 53 ms delay conditions. Only the peaks that belonged to clusters with a size greater than four voxels are reported for spatial restrictions. Download Table 6-2, DOCX file.

## Discussion

Computational theories have proposed that the brain uses internal forward models and information from our motor commands to predict the timing of sensory consequences of our movements and to attenuate sensory input presented at that specific time (Blakemore et al., 2000a; Wolpert and Flanagan, 2001; Bays and Wolpert, 2008). In contrast to previous neuroimaging studies imposing large temporal perturbations and thus contrasting somatosensory reafference with exafference conditions (Blakemore et al., 2001; Shergill et al., 2013), the present study focused on comparing conditions of somatosensory reafference with or without a brief temporal perturbation (i.e., 153 ms). Specifically, we compared trials where the touch was delivered close to its expected timing (i.e., with the 53 ms delay) previously shown to not have an impact on predictive mechanisms and simulate self-touch (Bays et al., 2005), with trials where the touch was delivered with a 153 ms temporal perturbation. This allowed us to test, for the first time, whether this time-locked predictive attenuation is disrupted when brief temporal perturbations are introduced between the predicted and actual times of the somatosensory reafference, as well as how somatosensory and motor connectivity changes in response to these perturbations.

At the perceptual level, we found that somatosensory reafference (i.e., self-generated touch) feels stronger when delivered with a 153 ms delay compared with when it is received close to its predicted timing (i.e., 53 ms). These perceptual effects were mirrored at the neural level; both the right primary and secondary somatosensory cortices showed increased activity when the self-generated touch is delivered with a 153 ms delay compared with when it is received close to its predicted timing (i.e., 53 ms). Importantly, the disruption of perceptual attenuation was significantly correlated with the disruption of the neural attenuation of the right primary somatosensory cortex; that is, participants who showed a larger effect of temporal perturbation on perception were the ones who showed a larger effect of temporal perturbation on somatosensory responses. These results suggest two novel conclusions. First, they demonstrate that somatosensory reafference is attenuated in both primary and secondary somatosensory cortices, in contrast to previous studies reporting effects only in the secondary somatosensory cortex when contrasting somatosensory reafference with exafference (Blakemore et al., 1998; Kilteni and Ehrsson, 2020). Straube et al., 2017, provides results in the visual/auditory domain. Given that the SI is the earliest processing node in the cortical somatosensory processing system (Kandel et al., 2000) and that the SII receives information from the S1 through ipsilateral corticocortical connections, our findings reveal that sensorimotor prediction has an impact on somatosensory processing earlier than previously thought. Second, these results reveal, for the first time, a direct relationship between perceptual and neural attenuation and suggest that the primary somatosensory cortex reflects the degree to which participants perceived somatosensory reafference, although touches had identical intensity (2 N) in both conditions.

In our univariate analysis, we observed increased activity in the cerebellum during temporal perturbations, consistent with other studies in the visual/auditory domain that reported cerebellar involvement in processing subtle delays (<100 ms) during self-generated movements (Arikan et al., 2019; Van Kemenade et al., 2019). In our study, this activity was localized to the right cerebellum, consistent with earlier PET findings (Blakemore et al., 2001), but not the left hemisphere. At first, the absence of left cerebellar activation might seem puzzling, given that the cerebellum contains ipsilateral body representations, and temporal perturbations relate to the touch applied on the left hand. A possible explanation can be the short duration of the temporal perturbation (i.e., 153 ms), but this is unlikely as Shergill et al. (2013) imposed a longer delay of 500 ms and did not observe left cerebellar activity either. Interestingly, a recent meta-analysis on the robustness of cerebellar activation during visual and auditory sensorimotor errors, including temporal perturbations, failed to detect consistent cerebellar activations across the examined studies (Johnson et al., 2019). The authors observed that cerebellar activations were most prominent in experiments where participants adapted to the imposed perturbation. In one of our previous studies (Kilteni et al., 2019), we showed that when repeatedly exposed to > 400 trials with 100 ms delays in somatosensory reafference, participants learn to predict the delayed touch and start to attenuate it. In contrast, in the present study, we purposefully included a few exposure trials to prevent such learning of 153 ms (50 trials in the behavioral task and 24 trials in each fMRI block); indeed, both behavioral and univariate control analyses showed that a short exposure to delays did not produce any significant learning of the temporal perturbation. Therefore, we speculate that this lack of adaptation can potentially explain the absence of left cerebellar effects.

Our functional connectivity analysis showed that the right primary somatosensory cortex had decreased connectivity with the supplementary motor area, the cerebellum, and the secondary somatosensory cortex during the temporal perturbations. Critically, this connectivity decrease was a function of the perceived amplitude of the touch; that is, participants who perceived a larger change in their perception because of the temporal perturbation also showed a larger effect of the temporal perturbation on somatosensory connectivity with the supplementary motor area and the cerebellum (i.e., a greater decrease in connectivity). Previous results contrasting somatosensory reafference with exafference reported increased connectivity between the cerebellum and somatosensory cortices with (nondelayed) self-generated input compared with externally generated input as a function of participant perception (Kilteni and Ehrsson, 2020); stronger attenuation of self-generated touches compared with externally generated touches yielded stronger somatosensory connectivity with the cerebellum during self-generated touches compared with externally generated touches. The present findings extend these previous results by contrasting self-generated touch conditions and showing that a 153 ms temporal perturbation in somatosensory reafference is sufficient to disrupt the corticocerebellar connectivity previously suggested to implement somatosensory attenuation (Kilteni and Ehrsson, 2020). This highlights the remarkable temporal precision of sensorimotor predictions, as a brief temporal error of 153 ms between the predicted and actual sensory reafference produced similar disruption in somatosensory attenuation as unpredicted sensory exafference.

Our perceptual, neural, and connectivity effects, when combined, strongly agree with the framework of an internal forward model that predictively attenuates self-generated input (Wolpert and Flanagan, 2001; McNamee and Wolpert, 2019). Accordingly, the left premotor cortices generate the right-hand motor command and the associated efference copy that is used by the cerebellum to predict the sensory consequences of the action, including the touch on the left index finger. The cerebellar prediction is used to attenuate the received somatosensory activity. However, when the sensory input is delayed, the somatosensory activity is not attenuated, and thus, the received touch feels stronger. This is exactly what we observed in our psychophysics task and in the univariate analysis. Moreover, the cerebellar prediction about the timing of sensory consequences based on the efference copy precedes the delayed sensory feedback, which leads to weaker interaction with somatosensory areas. In line with this framework, our connectivity patterns showed a decrease in the connectivity between the primary and secondary somatosensory cortices (sensory feedback), cerebellum (forward model), and SMA (efference copy).

During the brief temporal perturbations, we observed that the SMA contralateral to the moving hand increased its connectivity with the cerebellum (lobules VIII). These findings assign a critical role to SMA connectivity for contrasting conditions of somatosensory reafference with and without subtle temporal perturbations. The SMA is the target of cerebellar projections (Akkal et al., 2007; Bostan and Strick, 2018), and its posterior part (the SMA proper) is connected to the corticospinal tract, precentral gyrus (M1), and ventrolateral thalamus (Johansen-Berg et al., 2004). Both the SMA and cerebellum are involved in temporal processing and temporal predictions (Rao et al., 1997; Ullén et al., 2003; Ivry and Schlerf, 2008; Wiener et al., 2010; Coull et al., 2011; Merchant and Yarrow, 2016), with the posterior SMA being particularly involved in sensorimotor subsecond temporal processing compared with the anterior SMA (Schwartze et al., 2012). The SMA is involved in motor planning and preparation (Passingham, 1993; Tanji and Shima, 1996; Makoshi et al., 2011; Ruan et al., 2018), and transcranial magnetic stimulation (TMS) over the SMA during voluntary movements produces perceptual effects consistent with disruption of the efference copy that allows the prediction and attenuation of somatosensory responses (Haggard and Whitford, 2004). Similarly, the cerebellum is considered to implement the forward model (Shadmehr et al., 2008; McNamee and Wolpert, 2019; Popa and Ebner, 2019), and cerebellar TMS produces perceptual effects consistent with disruption of sensorimotor prediction and its combination with actual sensory feedback (Miall et al., 2007). From a theoretical perspective, functional connectivity between the SMA and cerebellum could refer to (1) the efference copy being sent to the cerebellar forward model to predict sensory consequences of the movement or (2) the error signal being sent back to the SMA to inform the motor centers about the errors. Our connectivity analysis is unable to distinguish between these two scenarios. However, given that efference copy-based sensorimotor predictions should be computed independently of temporal perturbations and that this connectivity increased during temporal perturbations, we propose that the most compatible interpretation is that of communicating the temporal prediction error.

Directly after the fMRI and psychophysics sessions mentioned above, we asked our participants whether they perceived the presence of any delay between the movement of their right hand and the somatosensory feedback on their left hand. Only one participant reported noticing the 153 ms delay. Similarly, in an earlier behavioral study, Blakemore et al. (1999) reported that delays up to 300 ms were not systematically detected by the participants. We speculate that the conscious detection of the temporal perturbation could have triggered activity in areas such as the angular gyrus that are related to conscious action feedback monitoring (Van Kemenade et al., 2019) and cross-modal asynchrony detection during movement (Abdulkarim et al., 2023), which we did not observe in our study. However, as neither of the previous neuroimaging studies using large somatosensory delays (Blakemore et al., 2001; Shergill et al., 2013), nor the present one systematically assessed whether participants perceived the temporal perturbation on a trial-to-trial basis, future studies are needed to examine the relationship between awareness of temporal delays in self-touch and the behavioral and neural effects of attenuation.

Disturbances in the attenuation of somatosensory reafference have been repeatedly reported in patients with schizophrenia (Blakemore et al., 2000b; Shergill et al., 2005, 2014) and nonclinical individuals high in schizotypal personality traits (Asimakidou et al., 2022). Straube et al. (2020) and Uhlmann et al. (2021) provide findings in other sensory modalities. Using encephalography, it was further shown that schizophrenic patients suppress their nondelayed self-generated sounds to a lesser extent than healthy controls but show normal attenuation when the auditory reafference is delayed (Whitford et al., 2011). We therefore theorize that the pattern of effects revealed by the present study might be reversed in such patients, leading to the attenuation of the delayed somatosensory reafference but not the nondelayed one. This hypothesis should be investigated in future experiments.
