# Supplementary figures and images for: Brief Temporal Perturbations in Somatosensory Reafference Disrupt Perceptual and Neural Attenuation and Increase Supplementary Motor Area–Cerebellar Connectivity
Source: J Neurosci. 2023 Jul 12;43(28):5251–63. doi: 10.1523/JNEUROSCI.1743-22.2023 (PMC10342225; doi:10.1523/JNEUROSCI.1743-22.2023)

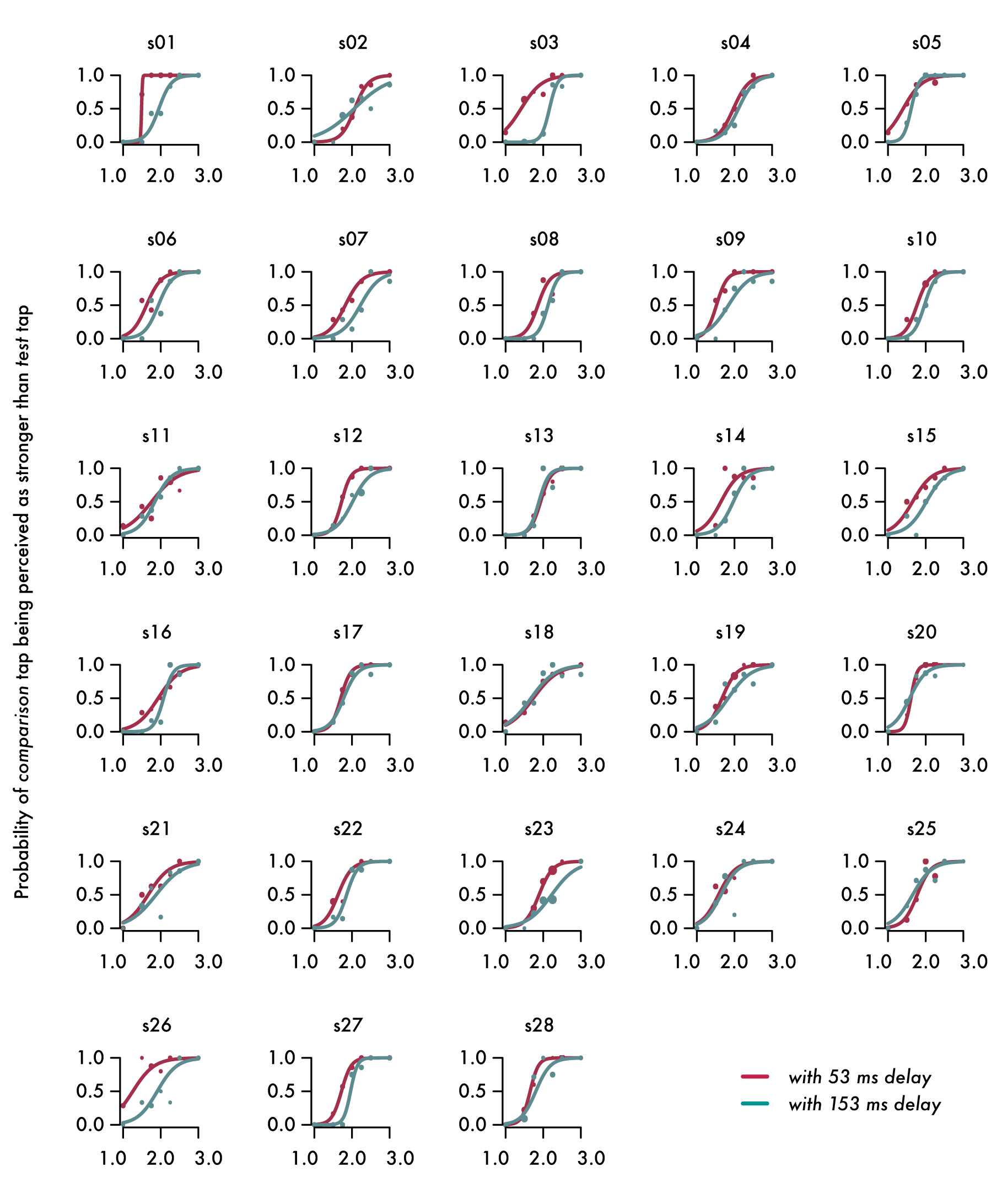

Supplement: Figure 3-1 — Individual plots of the psychophysical session. The marker size is proportional to the number of repetitions at that stimulus level. For all participants and conditions, the fitted model resulted in a McFadden’s R2 value ranging between 0.409 and 0.945. Download Figure 3-1, TIF file. [file ns-JN-RM-1743-22-s01.tif]

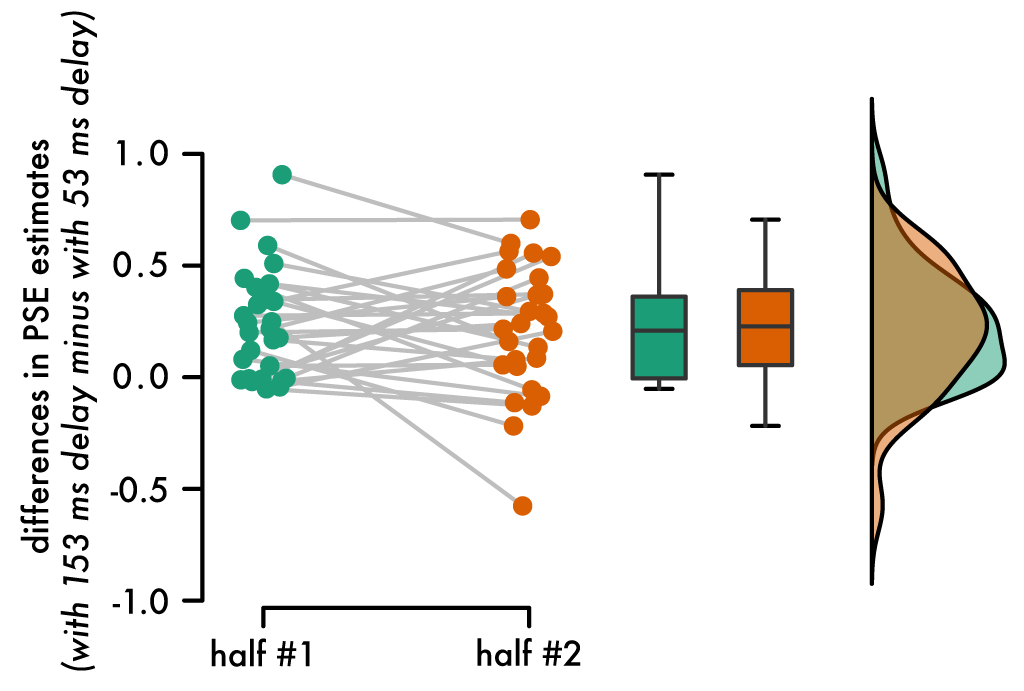

Supplement: Figure 3-2 — Absence of learning effects of the 153 ms delay during the psychophysical session. Individual differences and line plots illustrating the difference in the PSE values of the two conditions (self-generated touch with the 153 ms delay – self-generated touch with the 53 ms delay self-generated touch) between the first and the second halves of the psychophysical task. Box plots and raincloud plots illustrate the group effects. There were no learning effects, as strongly supported by a Bayesian analysis. Download Figure 3-2, TIF file. [file ns-JN-RM-1743-22-s02.tif]

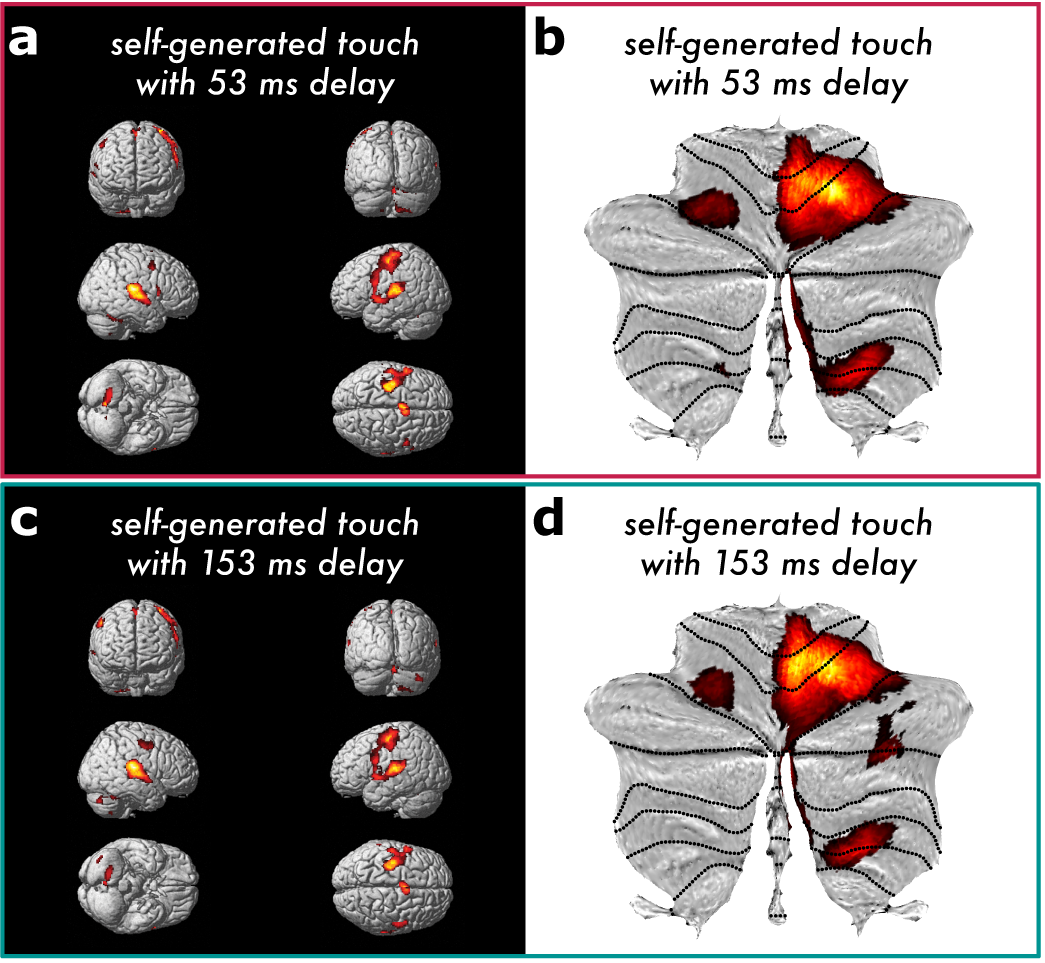

Supplement: Figure 4-1 — Activations during the self-generated touch with the 53 ms delay and self-generated touch with the 153 ms delay conditions. a, b, Activations reflect greater effects of the self-generated touch with the 53 ms delay condition than the rest condition. c, d, Activations reflect greater effects during the self-generated touch with the 153 ms delay condition than the rest condition. In both contrasts, auditory areas were also activated because the participants heard auditory GO cues to produce the self-generated touches. The activations were rendered on the standard single-subject 3D volume provided with SPM (a, c). Cerebellar activations were overlaid onto a cerebellar flat map (b, d). All activation maps (a–d) are displayed at a threshold of p < 0.05, FWE corrected. Download Figure 4-1, TIF file. [file ns-JN-RM-1743-22-s03.tif]

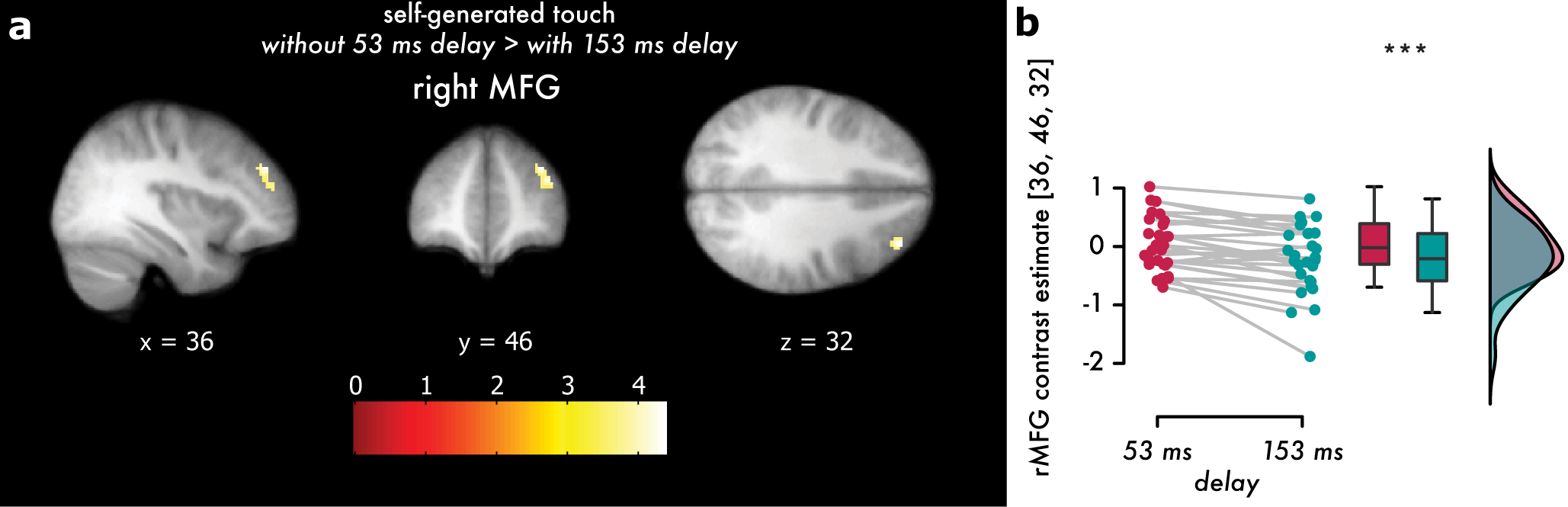

Supplement: Figure 4-2 — Activations elicited during the self-generated touch with the 53 ms delay compared with the self-generated touch with the 153 ms delay conditions. a, Activations reflect greater effects during the self-generated touch with the 53 ms delay compared with the self-generated touch with the 153 ms delay in the right middle frontal gyrus that did not survive corrections for multiple comparisons. The activations are rendered on the mean structural image across all participants. All activation maps are displayed at a threshold of p < 0.001 uncorrected (Exended Data Table 4-4). b, Individual contrast estimates and line plots illustrating the increase in the activation of the middle frontal gyrus in the self-generated touch with the 53 ms delay compared with the self-generated touch with the 153 ms delay conditions (p < 0.001). Download Figure 4-2, TIF file. [file ns-JN-RM-1743-22-s04.tif]

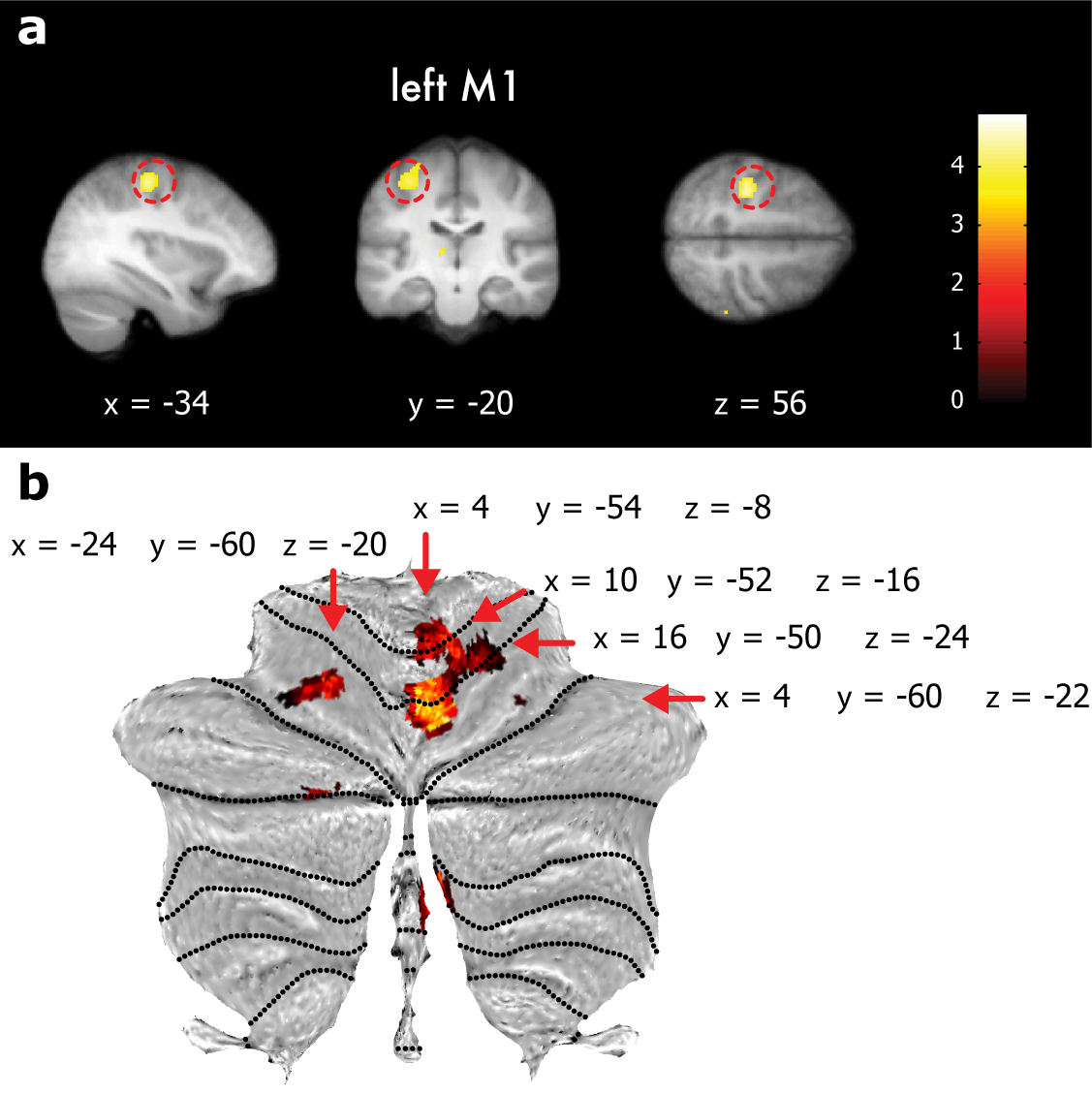

Supplement: Figure 4-3 — a, b, Sensorimotor (a) and cerebellar (b) areas whose BOLD activity was significantly and linearly modulated by the forces participants exerted with their right index finger (active taps). The activity of the left motor cortex was significantly modulated by the strength of the active taps (a). The cluster extends to the left primary somatosensory cortex. The red circle indicates the significant peak. The activations were rendered on the mean structural image across all participants at an uncorrected threshold of p < 0.001. Multiple peaks in the right and left cerebellum (b) were significantly modulated by the taps of the participants’ right hand (lobules V, VI). Arrows denote the significant peaks. The cerebellar activations were rendered on the cerebellar flat map at an uncorrected threshold of p < 0.001. Download Figure 4-3, TIF file. [file ns-JN-RM-1743-22-s05.tif]

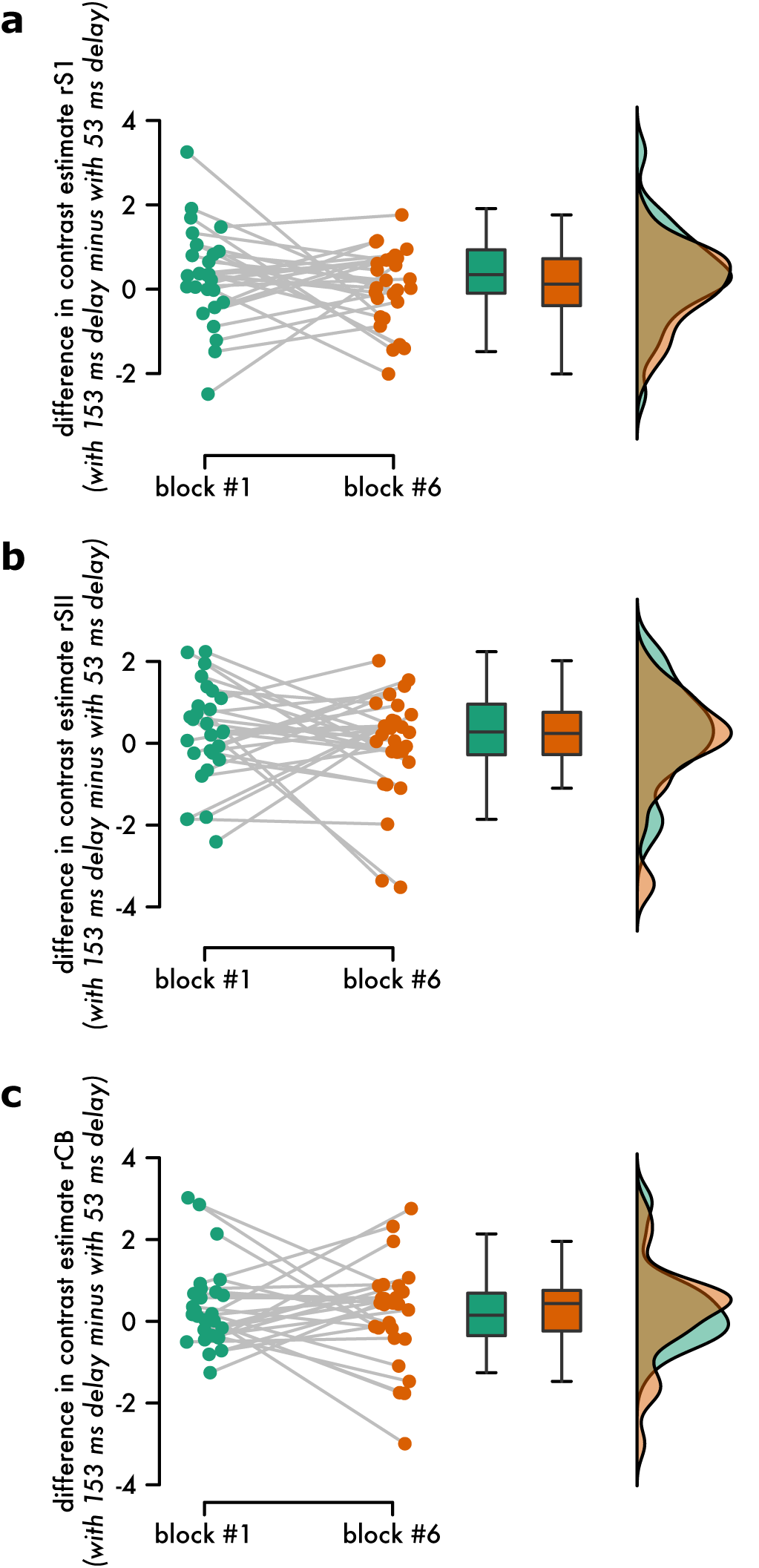

Supplement: Figure 4-4 — Absence of learning effects of the 153 ms delay during the fMRI run. a–c, Individual differences and line plots illustrating the difference in the extracted activity for contrast estimates between the two conditions (self-generated touch with the 153 ms delay – self-generated touch with the 53 ms delay) between the first and the last block of the fMRI run in the (a) right primary somatosensory cortex (rS1), (b) secondary somatosensory cortex (rSII), and (c) the right cerebellum (rCB). Box plots and raincloud plots illustrate the group effects. There were no learning effects, as strongly supported by a Bayesian analysis. Download Figure 4-4, TIF file. [file ns-JN-RM-1743-22-s06.tif]
