# Supplementary material for: Brief Temporal Perturbations in Somatosensory Reafference Disrupt Perceptual and Neural Attenuation and Increase Supplementary Motor Area–Cerebellar Connectivity
Source: J Neurosci. 2023 Jul 12;43(28):5251–63. doi: 10.1523/JNEUROSCI.1743-22.2023 (PMC10342225; doi:10.1523/JNEUROSCI.1743-22.2023)
Supplement: Table 4-1 — Activation peaks for the self-generated touch with the 53 ms delay condition. Peaks reflect greater effects during self-generated touch with the 53 ms delay compared with rest (self-generated touch with the 53 ms delay >0). Only the peaks that survived the FWE correction (p < 0.05) belonging to clusters with a size > four voxels are reported for spatial restrictions. Download Table 4-1, DOCX file. [file ns-JN-RM-1743-22-s07.docx]

**Table 4-1. Activation peaks for the *self-generated touch with*** ***the 53 ms delay*** **condition.** Peaks﻿ reflect greater effects during *self-generated touch with the 53 ms delay* compared to rest (*self-generated touch with the 53 ms delay* > 0). Only the peaks that survived the FWE correction (*p* < 0.05) belonging to clusters with size greater than 4 voxels are reported for spatial restrictions.

| Brain region | Cluster size (voxels) | MNI coordinates (mm) | | | *z* | *p* |
| --- | --- | --- | --- | --- | --- | --- |
|  |  | x | y | z |  |  |
| R cerebellum VI (Hem) | 1990 | 20 | -54 | -26 | Inf | *p* < 0.001 *FWE-corrected* |
| R cerebellum V (Hem) |  | 8 | -54 | -16 | 7.29 | *p* < 0.001 *FWE-corrected* |
| R cerebellum VIIIb (Hem) |  | 20 | -60 | -50 | 6.31 | *p* < 0.001 *FWE-corrected* |
| R cerebellum VI (Hem) |  | 18 | -68 | -22 | 5.60 | *p* < 0.001 *FWE-corrected* |
| R cerebellum VIIIa (Vermis) |  | 6 | -66 | -36 | 5.42 | *p* < 0.001 *FWE-corrected* |
| L precentral gyrus (M1) | 3299 | -32 | -24 | 54 | 6.76 | *p* < 0.001 *FWE-corrected* |
| L precentral gyrus |  | -60 | 6 | 24 | 6.58 | *p* < 0.001 *FWE-corrected* |
| L parietal operculum (SII) |  | -52 | -22 | 12 | 6.42 | *p* < 0.001 *FWE-corrected* |
| L parietal operculum (SII) |  | -40 | -30 | 18 | 6.41 | *p* < 0.001 *FWE-corrected* |
| L precentral gyrus |  | -58 | 4 | 32 | 6.35 | *p* < 0.001 *FWE-corrected* |
| L precentral gyrus |  | -38 | -16 | 54 | 6.30 | *p* < 0.001 *FWE-corrected* |
| L postcentral gyrus (S1) |  | -58 | -16 | 46 | 6.20 | *p* < 0.001 *FWE-corrected* |
| L parietal operculum |  | -40 | -4 | 12 | 6.08 | *p* < 0.001 *FWE-corrected* |
| L postcentral gyrus (S1) |  | -48 | -12 | 58 | 6.02 | *p* < 0.001 *FWE-corrected* |
| L superior temporal gyrus |  | -40 | -24 | 2 | 5.94 | *p* < 0.001 *FWE-corrected* |
| L precentral gyrus |  | -38 | -26 | 68 | 5.90 | *p* < 0.001 *FWE-corrected* |
| L postcentral gyrus (S1) |  | -52 | -12 | 52 | 5.73 | *p* < 0.001 *FWE-corrected* |
| L parietal operculum |  | -52 | 8 | 2 | 5.71 | *p* < 0.001 *FWE-corrected* |
| L superior temporal gyrus |  | -48 | -8 | -4 | 5.60 | *p* < 0.001 *FWE-corrected* |
| L superior temporal gyrus |  | -54 | 4 | -2 | 5.56 | *p* = 0.001 *FWE-corrected* |
| L inferior frontal gyrus (pars opercularis) |  | -56 | 6 | 12 | 5.18 | *p* = 0.003 *FWE-corrected* |
| R superior temporal gyrus | 1748 | 56 | -14 | 2 | 6.71 | *p* < 0.001 *FWE-corrected* |
| R superior temporal gyrus |  | 62 | -26 | 14 | 6.66 | *p* < 0.001 *FWE-corrected* |
| R Heschl’s gyrus |  | 40 | -24 | 6 | 5.89 | *p* < 0.001 *FWE-corrected* |
| R parietal operculum |  | 38 | -30 | 20 | 5.86 | *p* < 0.001 *FWE-corrected* |
| R parietal operculum (SII) |  | 44 | -30 | 18 | 5.83 | *p* < 0.001 *FWE-corrected* |
| R superior temporal gyrus |  | 50 | -4 | -6 | 5.82 | *p* < 0.001 *FWE-corrected* |
| R insula |  | 42 | 0 | -2 | 4.83 | *p* = 0.016 *FWE-corrected* |
| L superior frontal gyrus (SMA) | 474 | -6 | -2 | 58 | 6.27 | *p* < 0.001 *FWE-corrected* |
| R superior frontal gyrus (SMA) |  | 2 | 0 | 60 | 6.11 | *p* < 0.001 *FWE-corrected* |
| L cerebellum VI (Hem) | 135 | -26 | -58 | -26 | 5.60 | *p* < 0.001 *FWE-corrected* |
| R precentral gyrus | 71 | 52 | 4 | 46 | 5.34 | *p* = 0.002 *FWE-corrected* |
| L thalamus | 26 | -14 | -20 | 6 | 5.07 | *p* = 0.006 *FWE-corrected* |
| R inferior frontal gyrus (pars opercularis) | 59 | 54 | 14 | 8 | 5.03 | *p* = 0.007 *FWE-corrected* |
| R precentral gyrus |  | 60 | 10 | 18 | 4.64 | *p* = 0.032 *FWE-corrected* |
| L cerebellum VIIIb (Hem) | 7 | -14 | -64 | -52 | 4.88 | *p* = 0.012 *FWE-corrected* |
