# Supplementary material for: Brief Temporal Perturbations in Somatosensory Reafference Disrupt Perceptual and Neural Attenuation and Increase Supplementary Motor Area–Cerebellar Connectivity
Source: J Neurosci. 2023 Jul 12;43(28):5251–63. doi: 10.1523/JNEUROSCI.1743-22.2023 (PMC10342225; doi:10.1523/JNEUROSCI.1743-22.2023)
Supplement: Table 4-2 — Activation peaks for the self-generated touch with the 153 ms delay condition. Peaks reflect greater effects during self-generated touch with the 153 ms delay compared with rest (self-generated touch with the 153 ms delay >0). Only the peaks that survived the FWE correction (p < 0.05) belonging to clusters with a size > four voxels are reported for spatial restrictions. Download Table 4-2, DOCX file. [file ns-JN-RM-1743-22-s08.docx]

**Table 4-2. Activation peaks for the *self-generated touch with the 153 ms delay* condition.** Peaks﻿ reflect greater effects during *self-generated touch with the 153 ms delay* compared to rest (*self-generated touch with the 153 ms delay* > 0). Only the peaks that survived the FWE correction (*p* < 0.05) belonging to clusters with size greater than 4 voxels are reported for spatial restrictions.

| Brain region | Cluster size (voxels) | MNI coordinates (mm) | | | *z* | *p* |
| --- | --- | --- | --- | --- | --- | --- |
|  |  | x | y | z |  |  |
| R cerebellum VI (Hem) | 1759 | 20 | -54 | -26 | 7.77 | *p* < 0.001 *FWE-corrected* |
| R cerebellum V (Hem) |  | 6 | -56 | -12 | 7.30 | *p* < 0.001 *FWE-corrected* |
| R cerebellum V (Hem) |  | 6 | -60 | -24 | 6.42 | *p* < 0.001 *FWE-corrected* |
| R cerebellum VIIIa (Vermis) |  | 8 | -66 | -38 | 4.93 | *p* = 0.009 *FWE-corrected* |
| R parietal operculum (SII) | 2003 | 56 | -24 | 16 | 6.94 | *p* < 0.001 *FWE-corrected* |
| R superior temporal gyrus |  | 56 | -16 | 4 | 6.24 | *p* < 0.001 *FWE-corrected* |
| R superior temporal gyrus |  | 50 | -12 | 0 | 6.22 | *p* < 0.001 *FWE-corrected* |
| R superior temporal gyrus |  | 62 | -24 | 6 | 6.12 | *p* < 0.001 *FWE-corrected* |
| R superior temporal gyrus |  | 48 | -6 | -2 | 5.98 | *p* < 0.001 *FWE-corrected* |
| L parietal operculum (SII) | 3292 | -54 | -24 | 12 | 6.78 | *p* < 0.001 *FWE-corrected* |
| L precentral gyrus |  | -60 | 6 | 26 | 6.66 | *p* < 0.001 *FWE-corrected* |
| L parietal operculum |  | -36 | -34 | 16 | 6.50 | *p* < 0.001 *FWE-corrected* |
| L precentral gyrus |  | -30 | -22 | 56 | 6.44 | *p* < 0.001 *FWE-corrected* |
| L parietal operculum |  | -40 | -4 | 12 | 6.41 | *p* < 0.001 *FWE-corrected* |
| L precentral gyrus |  | -38 | -14 | 56 | 6.39 | *p* < 0.001 *FWE-corrected* |
| L postcentral gyrus (S1) |  | -56 | -16 | 46 | 6.22 | *p* < 0.001 *FWE-corrected* |
| L temporal pole |  | -52 | 6 | 0 | 5.81 | *p* < 0.001 *FWE-corrected* |
| L superior temporal gyrus |  | -40 | -24 | 2 | 5.67 | *p* < 0.001 *FWE-corrected* |
| L inferior frontal gyrus (pars opercularis) |  | -58 | 6 | 12 | 5.38 | *p* = 0.001 *FWE-corrected* |
| L superior temporal gyrus |  | -64 | -38 | 18 | 5.35 | *p* = 0.001 *FWE-corrected* |
| L precentral gyrus |  | -48 | 0 | 52 | 5.32 | *p* = 0.002 *FWE-corrected* |
| L superior temporal gyrus |  | -50 | -6 | -2 | 5.12 | *p* = 0.004 *FWE-corrected* |
| L Heschl’s gyrus |  | -34 | -28 | 6 | 4.94 | *p* = 0.009 *FWE-corrected* |
| L precentral gyrus |  | -54 | 4 | 40 | 4.88 | *p* = 0.012 *FWE-corrected* |
| L precentral gyrus |  | -50 | -2 | 44 | 4.80 | *p* = 0.016 *FWE-corrected* |
| R superior frontal gyrus (SMA) | 378 | 2 | 2 | 60 | 6.13 | *p* < 0.001 *FWE-corrected* |
| L superior frontal gyrus (SMA) |  | -6 | -2 | 58 | 6.07 | *p* < 0.001 FWE-corrected |
| R cerebellum VIIIb (Hem) | 176 | 20 | -60 | -50 | 5.98 | *p* < 0.001 *FWE-corrected* |
| R cerebellum VIIa Crus I (Hem) | 75 | 44 | -74 | -36 | 5.46 | *p* = 0.001 *FWE-corrected* |
| R cerebellum VIIa Crus I (Hem) |  | 38 | -72 | -30 | 4.69 | *p* = 0.026 *FWE-corrected* |
| R cerebellum VIIa Crus I (Hem) |  | 44 | -62 | -30 | 4.61 | *p* = 0.035 *FWE-corrected* |
| R precentral gyrus | 179 | 52 | 4 | 48 | 5.39 | *p* = 0.001 *FWE-corrected* |
| R precentral gyrus |  | 56 | -8 | 48 | 5.39 | *p* = 0.001 *FWE-corrected* |
| L cerebellum VI (Hem) | 84 | -24 | -58 | -26 | 5.27 | *p* = 0.002 *FWE-corrected* |
