# Supplementary material for: Brief Temporal Perturbations in Somatosensory Reafference Disrupt Perceptual and Neural Attenuation and Increase Supplementary Motor Area–Cerebellar Connectivity
Source: J Neurosci. 2023 Jul 12;43(28):5251–63. doi: 10.1523/JNEUROSCI.1743-22.2023 (PMC10342225; doi:10.1523/JNEUROSCI.1743-22.2023)
Supplement: Table 4-3 — Activations greater during the self-generated touch with the 153 ms delay than the self-generated touch with the 53 ms delay conditions. Peaks reflect greater effects of the self-generated touch with the 153 ms delay compared with the self-generated touch with the 53 ms delay conditions. Download Table 4-3, DOCX file. [file ns-JN-RM-1743-22-s09.docx]

**Table 4-3. Activations greater during the *self-generated touch with the 153 ms delay* than the *self-generated touch with the 53 ms delay* conditions.** Peaks﻿ reflect greater effects of the *self-generated touch with the 153 ms delay* compared to the *self-generated touch with the 53 ms delay* conditions.

| Brain region | Cluster size (voxels) | MNI coordinates (mm) | | | *z* | *p* |
| --- | --- | --- | --- | --- | --- | --- |
|  |  | x | y | z |  |  |
| R parietal operculum | 86 | 50 | -30 | 20 | 4.33 | *p* < 0.001 *uncorrected* |
| R postcentral gyrus (S1) | 59^1^ | 48 | -18 | 60 | 4.07 | *p* = 0.002 *FWE-corrected* ^*^ |
| R postcentral gyrus (S1) |  | 50 | -16 | 56 | 3.98 | *p* = 0.002 *FWE-corrected* ^*^ |
| R precentral/postcentral gyrus |  | 54 | -12 | 46 | 3.69 | *p* < 0.001 *uncorrected* |
| L superior frontal gyrus | 72 | -12 | 44 | 30 | 3.99 | *p* < 0.001 *uncorrected* |
| L inferior frontal gyrus (pars orbitalis) | 39 | -46 | 34 | -10 | 3.72 | *p* < 0.001 *uncorrected* |
| L inferior frontal gyrus (pars triangularis) | 91 | -40 | 22 | 22 | 3.72 | *p* < 0.001 *uncorrected* |
| L inferior frontal gyrus (pars triangularis) |  | -48 | 20 | 16 | 3.38 | *p* < 0.001 *uncorrected* |
| L inferior frontal gyrus (pars triangularis) |  | -54 | 26 | 10 | 3.24 | *p* = 0.001 *uncorrected* |
| R parietal operculum (SII) | 54^2^ | 42 | -20 | 16 | 3.71 | *p* = 0.006 *FWE-corrected* ^*^ |
| R hippocampus | 12 | 36 | -16 | -16 | 3.64 | *p* < 0.001 *uncorrected* |
| R cerebellum VIIa Crus I (Hem) | 49 | 36 | -72 | -34 | 3.57 | *p* = 0.049 *FWE-corrected* ^*^ |
| R middle cingulate gyrus | 11 | 14 | -20 | 46 | 3.52 | *p* < 0.001 *uncorrected* |
| L middle frontal gyrus | 24 | -34 | 12 | 46 | 3.48 | *p* < 0.001 *uncorrected* |
| L middle temporal gyrus | 30 | -54 | -18 | -18 | 3.44 | *p* < 0.001 *uncorrected* |
| L inferior parietal lobule | 11 | -32 | -72 | 42 | 3.36 | *p* < 0.001 *uncorrected* |
| L supramarginal gyrus | 14 | -48 | -46 | 26 | 3.35 | *p* < 0.001 *uncorrected* |
| R postcentral gyrus | 8 | 60 | -2 | 36 | 3.32 | *p* < 0.001 *uncorrected* |
| R insula | 12 | 30 | -20 | 16 | 3.31 | *p* < 0.001 *uncorrected* |

**^*^** After small-volume correction.

^1^ The cluster size was 106 before corrections for multiple comparisons and was reduced to 59 after small-volume correction.

^2^ The cluster size was 74 before corrections for multiple comparisons and was reduced to 54 after small-volume correction.
