# Supplementary material for: Brief Temporal Perturbations in Somatosensory Reafference Disrupt Perceptual and Neural Attenuation and Increase Supplementary Motor Area–Cerebellar Connectivity
Source: J Neurosci. 2023 Jul 12;43(28):5251–63. doi: 10.1523/JNEUROSCI.1743-22.2023 (PMC10342225; doi:10.1523/JNEUROSCI.1743-22.2023)
Supplement: Table 4-4 — Activations greater during the self-generated touch with the 53 ms delay than the self-generated touch with the 153 ms delay conditions. Peaks reflect greater effects of the self-generated touch with the 53 ms delay compared with self-generated touch with the 153 ms delay conditions. Download Table 4-4, DOCX file. [file ns-JN-RM-1743-22-s10.docx]

**Table 4-4. Activations greater during the *self-generated touch with the 53 ms delay* than the *self-generated touch with the 153 ms delay* conditions.** Peaks﻿ reflect greater effects of the *self-generated touch with the 53 ms delay* compared to *self-generated touch with the 153 ms delay* conditions.

| Brain region | Cluster size (voxels) | MNI coordinates (mm) | | | *z* | *p* |
| --- | --- | --- | --- | --- | --- | --- |
|  |  | x | y | z |  |  |
| R middle frontal gyrus | 95 | 36 | 46 | 32 | 3.78 | *p* < 0.001 *uncorrected* |
| R middle frontal gyrus |  | 44 | 44 | 22 | 3.71 | *p* < 0.001 *uncorrected* |
| R middle frontal gyrus |  | 36 | 50 | 20 | 3.32 | *p* < 0.001 *uncorrected* |
| R inferior parietal lobule | 18 | 60 | -34 | 46 | 3.41 | *p* < 0.001 *uncorrected* |
