# Supplementary material for: Brief Temporal Perturbations in Somatosensory Reafference Disrupt Perceptual and Neural Attenuation and Increase Supplementary Motor Area–Cerebellar Connectivity
Source: J Neurosci. 2023 Jul 12;43(28):5251–63. doi: 10.1523/JNEUROSCI.1743-22.2023 (PMC10342225; doi:10.1523/JNEUROSCI.1743-22.2023)
Supplement: Table 4-5 — Areas whose activity was parametrically modulated by the strength of the right-hand active taps across the self-generated touch with the 53 ms and the 153 ms delay conditions. Download Table 4-5, DOCX file. [file ns-JN-RM-1743-22-s11.docx]

**Table 4-5. Areas whose activity was parametrically modulated by the strength of the right hand’s *active* taps across the *self-generated touch with 53 ms* and** ***the 153 ms delay*** **conditions.**

| Brain region | Cluster size (voxels) | MNI coordinates (mm) | | | *z* | *p* |
| --- | --- | --- | --- | --- | --- | --- |
|  |  | x | y | z |  |  |
| R cerebellum V (Hem) | 154^1^ | 4 | -60 | -22 | 4.09 | *p* = 0.004 *FWE-corrected* ^*^ |
| R cerebellum V (Hem) |  | 4 | -54 | -8 | 3.48 | *p* = 0.028 *FWE-corrected* ^*^ |
| R cerebellum V (Hem) |  | 10 | -52 | -16 | 3.46 | *p* = 0.030 *FWE-corrected* ^*^ |
| R cerebellum VI (Hem) |  | 8 | -64 | -14 | 3.36 | *p* < 0.001 *uncorrected* |
| R cerebellum V (Hem) |  | 16 | -50 | -24 | 3.34 | *p* = 0.043 *FWE-corrected* ^*^ |
| L precentral gyrus | 21^2^ | -34 | -20 | 56 | 3.51 | *p* = 0.011 *FWE-corrected* ^*^ |
| L precentral gyrus |  | -28 | -28 | 64 | 3.52 | *p* < 0.001 uncorrected |
| L cerebellum VI (Hem) | 32 | -24 | -60 | -20 | 3.53 | *p* = 0.043 *FWE-corrected* ^*^ |
| R cerebellum VIIIa (Vermis) | 16 | 6 | -68 | -36 | 3.45 | *p* < 0.001 *uncorrected* |
| L thalamus | 16 | -10 | -16 | 8 | 3.41 | *p* < 0.001 *uncorrected* |
| R superior frontal gyrus | 25 | 4 | 24 | 44 | 3.34 | *p* < 0.001 *uncorrected* |
| R cerebellum dentate nucleus | 23 | -14 | -68 | -34 | 3.30 | *p* < 0.001 *uncorrected* |
| R superior frontal gyrus | 5 | 16 | 16 | 62 | 3.23 | *p* = 0.001 *uncorrected* |
| R inferior parietal lobule | 6 | 54 | -38 | 54 | 3.19 | *p* = 0.001 *uncorrected* |

**^*^** After small-volume correction.

^1^ The cluster size was 283 before corrections for multiple comparisons and was reduced to 154 after small-volume correction

^2^ The cluster size was 297 before corrections for multiple comparisons and was reduced to 21 after small-volume correction.
