# Supplementary material for: Brief Temporal Perturbations in Somatosensory Reafference Disrupt Perceptual and Neural Attenuation and Increase Supplementary Motor Area–Cerebellar Connectivity
Source: J Neurosci. 2023 Jul 12;43(28):5251–63. doi: 10.1523/JNEUROSCI.1743-22.2023 (PMC10342225; doi:10.1523/JNEUROSCI.1743-22.2023)
Supplement: Table 5-1 — Peaks with decreased connectivity with the left primary somatosensory cortex during the temporal perturbation as a function of the PSE difference between the self-generated touch with the 153 ms delay and self-generated touch with the 53 ms delay conditions. Peaks reflect reduced connectivity with the left primary somatosensory cortex in the self-generated touch with the 153 ms delay compared with the self-generated touch with the 53 ms delay conditions and covaried with participant perception. Only the peaks that belonged to clusters with a size greater than four voxels are reported for spatial restrictions. Download Table 5-1, DOCX file. [file ns-JN-RM-1743-22-s13.docx]

**Table 5-1. Peaks with decreased connectivity with the left primary somatosensory cortex during the temporal perturbation as a function of the PSE difference between the *self-generated touch with*** ***the 153 ms delay* and *self-generated touch with*** ***the 53 ms delay*** **conditions.** Peaks reflect reduced connectivity with the left primary somatosensory cortex in the *self-generated touch with the 153 ms delay* compared to the *self-generated touch with the 53 ms delay* conditions and covaried with participant perception. Only the peaks that belonged to clusters with size greater than 4 voxels are reported for spatial restrictions.

| Brain region | Cluster size (voxels) | MNI coordinates (mm) | | | *z* | *p* |
| --- | --- | --- | --- | --- | --- | --- |
|  |  | x | y | z |  |  |
| L cerebellum VIIIa (Hem) | 25^1^ | -30 | -44 | -58 | 4.54 | *p* = 0.001 *FWE-corrected** |
| L precuneus | 120 | -12 | -68 | 34 | 4.52 | *p* < 0.001 *uncorrected* |
| L parahippocampal gyrus | 29 | -14 | -8 | -26 | 4.31 | *p* < 0.001 *uncorrected* |
| L inferior occipital gyrus | 60 | -52 | -74 | -6 | 4.30 | *p* < 0.001 *uncorrected* |
| R precuneus | 83 | 10 | -68 | 32 | 4.17 | *p* < 0.001 *uncorrected* |
| L cerebellum VIIa Crus I (Hem) | 19 | -48 | -44 | -48 | 4.17 | *p* < 0.001 *uncorrected* |
| R superior orbital gyrus | 64 | 14 | 38 | -26 | 3.78 | *p* < 0.001 *uncorrected* |
| L superior frontal gyrus (SMA) | 72^2^ | -2 | -2 | 52 | 3.74 | *p* < 0.01 *FWE-corrected** |
| R fusiform gyrus | 20 | 32 | -24 | -30 | 3.69 | *p* < 0.001 *uncorrected* |
| L cerebellum VIIa Crus II | 24 | -46 | -58 | -52 | 3.59 | *p* < 0.001 *uncorrected* |
| L superior parietal lobule | 47 | -28 | -44 | 72 | 3.58 | *p* < 0.001 *uncorrected* |
| R cerebellum VIIIb (Hem) | 23 | 22 | -48 | -58 | 3.57 | *p* = 0.029 *FWE-corrected** |
| R precuneus | 100 | 12 | -68 | 58 | 3.56 | *p* < 0.001 *uncorrected* |
| L calcarine gyrus | 29 | 0 | -62 | 10 | 3.53 | *p* < 0.001 *uncorrected* |
| L cerebellum VIIIb (Hem) | 4^3^ | -16 | -62 | -60 | 3.39 | *p* = 0.043 *FWE-corrected** |
| R inferior occipital gyrus | 15 | 46 | -82 | -8 | 3.48 | *p* < 0.001 *uncorrected* |
| L Heschl’s gyrus | 18 | -54 | -10 | 10 | 3.48 | *p* < 0.001 *uncorrected* |
| R parietal operculum (SII) | 5 | 46 | -16 | 24 | 3.46 | *p* = 0.023 *FWE-corrected** |
| R cerebellum VIIIa | 9^4^ | 20 | -62 | -60 | 3.42 | *p* = 0.049 *FWE-corrected** |
| R parietal operculum | 15 | 48 | -6 | 8 | 3.38 | *p* < 0.001 *uncorrected* |
| R medial temporal gyrus | 7 | 14 | 0 | -26 | 3.36 | *p* < 0.001 *uncorrected* |
| L insula | 9 | -38 | -20 | 12 | 3.35 | *p* < 0.001 *uncorrected* |
| R parahippocampal gyrus | 6 | 20 | -6 | -32 | 3.32 | *p* < 0.001 *uncorrected* |
| L middle cingulate cortex | 6 | -8 | -14 | 32 | 3.30 | *p* < 0.001 *uncorrected* |
| L cingulate gyrus | 5 | -18 | -34 | 46 | 3.24 | *p* = 0.001 *uncorrected* |
| R subcallosal gyrus | 4 | 12 | 12 | -22 | 3.24 | *p* = 0.001 *uncorrected* |
| R inferior parietal lobule | 8 | 46 | -40 | 48 | 3.24 | *p* = 0.001 *uncorrected* |
| R middle cingulate cortex | 4 | 4 | -36 | 44 | 3.17 | *p* = 0.001 *uncorrected* |

**^*^** After small-volume correction.

^1^ The cluster size was 41 before corrections for multiple comparisons and was reduced to 25 after small-volume correction

^2^ The cluster size was 104 before corrections for multiple comparisons and was reduced to 72 after small-volume correction

^3^ The cluster size was 12 before corrections for multiple comparisons and was reduced to 4 after small-volume correction.

^4^ The cluster size was 18 before corrections for multiple comparisons and was reduced to 9 after small-volume correction.
