# Supplementary material for: Brief Temporal Perturbations in Somatosensory Reafference Disrupt Perceptual and Neural Attenuation and Increase Supplementary Motor Area–Cerebellar Connectivity
Source: J Neurosci. 2023 Jul 12;43(28):5251–63. doi: 10.1523/JNEUROSCI.1743-22.2023 (PMC10342225; doi:10.1523/JNEUROSCI.1743-22.2023)
Supplement: Table 6-1 — Peaks with increased connectivity with the left supplementary motor area during temporal perturbation. Peaks reflect greater connectivity with the left supplementary motor area in the self-generated touch with the 153 ms delay compared with the self-generated touch with the 53 ms delay conditions. Only the peaks that belonged to clusters with a size greater than four voxels are reported for spatial restrictions. Download Table 6-1, DOCX file. [file ns-JN-RM-1743-22-s14.docx]

**Table 6-1. Peaks with increased connectivity with the left supplementary motor area during temporal perturbation.** Peaks reflect greater connectivity with the left supplementary motor area in the *self-generated touch with the 153 ms delay* compared to the *self-generated touch with the 53 ms delay* conditions. Only the peaks that belonged to clusters with size greater than 4 voxels are reported for spatial restrictions.

| Brain region | Cluster size (voxels) | MNI coordinates (mm) | | | *z* | *p* |
| --- | --- | --- | --- | --- | --- | --- |
|  |  | x | y | z |  |  |
| R superior medial frontal gyrus | 107 | 12 | 68 | 2 | 4.40 | *p* < 0.001 *uncorrected* |
| R superior medial frontal gyrus |  | 2 | 68 | 6 | 3.44 | *p* < 0.001 *uncorrected* |
| L cerebellum VI (Hem) | 107^1^ | -16 | -62 | -18 | 4.39 | *p* = 0.004 *FWE-corrected** |
| L cerebellum VI (Hem) | 84^2^ | -34 | -48 | -36 | 4.22 | *p* = 0.007 *FWE-corrected** |
| L cerebellum VI (Hem) |  | -28 | -44 | -24 | 3.60 | *p* < 0.001 *uncorrected* |
| R middle temporal gyrus | 25 | 56 | -50 | 0 | 3.84 | *p* < 0.001 *uncorrected* |
| L cerebellum X (Hem) | 115 | -16 | -36 | -46 | 3.78 | *p* < 0.001 *uncorrected* |
| L cerebellum VIIIb (Hem) |  | -18 | -44 | -56 | 3.76 | *p* = 0.013 *FWE-corrected** |
| L cerebellum VIIIb (Hem) |  | -16 | -44 | -52 | 3.75 | *p* = 0.014 *FWE-corrected** |
| L cerebellum VIIIb (Hem) |  | -18 | -42 | -48 | 3.64 | *p* = 0.019 *FWE-corrected** |
| L lingual gyrus | 45 | -12 | -90 | -16 | 3.71 | *p* < 0.001 *uncorrected* |
| L cerebellum VIIIa (Hem) | 32^3^ | -28 | -58 | -52 | 3.69 | *p* = 0.019 *FWE-corrected** |
| L cerebellum IX (Hem) | 43 | -2 | -48 | -40 | 3.62 | *p* < 0.001 *uncorrected* |
| R/L superior frontal gyrus | 38 | 0 | -20 | 72 | 3.57 | *p* < 0.001 *uncorrected* |
| R fusiform gyrus | 52 | 22 | -48 | -14 | 3.54 | *p* < 0.001 *uncorrected* |
| R fusiform gyrus |  | 28 | -50 | -20 | 3.49 | *p* < 0.001 *uncorrected* |
| L cerebellum VIII (dentate nucleus) | 22 | -10 | -62 | -38 | 3.52 | *p* < 0.001 *uncorrected* |
| R lingual gyrus | 9 | 20 | -64 | -10 | 3.33 | *p* < 0.001 *uncorrected* |
| R cerebellum IX (Hem) | 11 | 6 | -54 | -38 | 3.32 | *p* < 0.001 *uncorrected* |
| L lingual gyrus | 15 | -16 | -70 | -10 | 3.29 | *p* = 0.001 *uncorrected* |
| R superior frontal gyrus | 4 | 22 | 12 | 58 | 3.21 | *p* = 0.001 *uncorrected* |

**^*^** After small-volume correction.

^1^ The cluster size was 109 before corrections for multiple comparisons and was reduced to 107 after small-volume correction

^2^ The cluster size was 123 before corrections for multiple comparisons and was reduced to 84 after small-volume correction

^3^ The cluster size was 33 before corrections for multiple comparisons and was reduced to 32 after small-volume correction.
