# Supplementary material for: Brief Temporal Perturbations in Somatosensory Reafference Disrupt Perceptual and Neural Attenuation and Increase Supplementary Motor Area–Cerebellar Connectivity
Source: J Neurosci. 2023 Jul 12;43(28):5251–63. doi: 10.1523/JNEUROSCI.1743-22.2023 (PMC10342225; doi:10.1523/JNEUROSCI.1743-22.2023)
Supplement: Table 6-2 — Peaks with decreased connectivity with the left supplementary motor area during temporal perturbations. Peaks reflect lower connectivity with the left supplementary motor area for the self-generated touch with the 153 ms delay compared with the self-generated touch with the 53 ms delay conditions. Only the peaks that belonged to clusters with a size greater than four voxels are reported for spatial restrictions. Download Table 6-2, DOCX file. [file ns-JN-RM-1743-22-s15.docx]

**Table 6-2. Peaks with decreased connectivity with the left supplementary motor area during temporal perturbations.** Peaks reflect lower connectivity with the left supplementary motor area for the *self-generated touch with the 153 ms delay* compared to the *self-generated touch with the 53 ms delay* conditions. Only the peaks that belonged to clusters with size greater than 4 voxels are reported for spatial restrictions.

| Brain region | Cluster size (voxels) | MNI coordinates (mm) | | | *z* | *p* |
| --- | --- | --- | --- | --- | --- | --- |
|  |  | x | y | z |  |  |
| R caudate nucleus | 16 | 18 | 22 | 10 | 3.28 | *p* = 0.001 *uncorrected* |
